# Co‐Production of Mycoprotein, Carotenoids, and B Vitamins From Cheese Whey by *Neurospora intermedia*

**DOI:** 10.1111/1750-3841.71294

**Published:** 2026-07-23

**Authors:** Farzaneh Dianatdar, Zahra Etemadifar, Mohammad J. Taherzadeh

**Affiliations:** ^1^ Department of Cell and Molecular Biology & Microbiology, Faculty of Biological Science and Technology University of Isfahan Isfahan Iran; ^2^ Swedish Centre for Resource Recovery University of Borås Borås Sweden; ^3^ Millow AB Gothenburg Sweden

**Keywords:** biorefinery, Box–Behnken design, filamentous fungi, mycoprotein, valorization

## Abstract

Cheese whey, a nutrient‐rich dairy byproduct that requires management due to its high organic load, was valorized using an integrated biorefinery platform with the edible fungus *Neurospora intermedia* to simultaneously produce mycoprotein, carotenoids, and B vitamins (B1, B2, B3, B6). Initial optimization employed a one‐factor‐at‐a‐time (OFAT) approach to evaluate the effects of whey concentration, initial pH, KNO_3_, salt supplementation, trace elements, precursor, and pellet‐inducing agents (CaCl_2_, glycerol, and Tween 80). Subsequent optimization via response surface methodology (RSM) revealed the limitations of OFAT analysis and identified optimal conditions for targeted outputs. Maximum biomass yield (7.8 g/L) was achieved at high whey concentration (40 g/L) and low pH (pH 4), whereas maximum nutrient yields, 249.96 µg/g carotenoids, 1.46 mg/g B1, 7.17 mg/g B2, 0.41 mg/g B3, and 1.2 mg/g B6, were attained at low whey concentration (10 g/L) and low pH (pH 4). These conditions also minimized pellet size to 2 mm, enhancing metabolite production. The process concurrently reduced whey's chemical oxygen demand by 25.8% and demonstrated high biosorption of metal ions (76.8% Ca, 69.9% Mg, 80.8% Fe, 62.5% K). CHNS mass balance analysis revealed carbon (19.8%), hydrogen (18.9%), nitrogen (40.3%), and sulfur (42.5%) transfer efficiencies from cheese whey to *N*. *intermedia* biomass, with a 2.24‐fold nitrogen enrichment (from 2.69% to 6.04%) corresponding to 37.8% crude protein and atomic ratios of C/N (7.84), N/S (36.2), H/C (1.85), and C/O (≈1.15). This study demonstrates the effective biorefining of whey into valuable products using RSM. This approach allows for the targeted production of high‐volume mycoprotein, nutrient‐dense extracts, or specific fungal morphology.

## 1 Introduction

With global cheese whey generation commonly estimated at ∼180–190 million tons per year, developing efficient and scalable biorefinery pathways for whey valorization is a critical industrial and ecological priority. A highly promising biorefinery strategy is the bioconversion of whey using microorganisms (Delgado‐Macuil et al. 2025). Filamentous fungi, particularly edible and food‐grade species, are renowned for exceptional metabolic versatility, rapid growth on complex substrates, and an innate capacity to synthesize a diverse array of valuable metabolites (Strong et al. 2022). Beyond the model organism *Neurospora intermedia*, several other edible filamentous fungi have demonstrated remarkable potential in similar biorefining different food wastes (Gmoser 2021). Species such as *Aspergillus oryzae*, a cornerstone of traditional Asian fermentations, *Rhizopus oligosporus* (used in tempeh production), and *Fusarium venenatum* (commercialized as Quorn mycoprotein) are all recognized for their efficient substrate utilization and protein‐rich biomass (Yashaswini et al. 2026). Notably, *N*. *intermedia* has been used in traditional food fermentations (e.g., oncom) and has been described as edible and generally regarded as safe (GRAS), celebrated for its rapid growth, high protein content, and natural proficiency in producing pigments like carotenoids and essential B‐complex vitamins. Its cultivation on whey offers a synergistic solution, simultaneously mitigating an environmental pollutant and generating nutritious fungal biomass (mycoprotein) biofortified with proteins, vitamins, and functional carotenoids (Radulescu et al. 2025).

Utilizing whey as a microbial cultivation medium efficiently channels its nutrients (lactose as the primary carbon source and whey proteins/peptides as organic nitrogen) directly into metabolism to fuel growth and target compound synthesis (El‐Aidie and Khalifa 2024). Critical interdependent parameters like pH, whey concentration, and inoculum size profoundly affect microbial physiology, morphology, and metabolite yield, making their optimization essential for maximizing titer, rate, and process economics (Malos et al. 2025). To efficiently navigate this complex multivariable space, statistical design of experiments (DoE) and response surface methodology (RSM) are indispensable, as they surpass traditional one‐factor‐at‐a‐time (OFAT) approaches by systematically elucidating factor interactions and nonlinear effects with minimal experimental runs, which is particularly powerful for modeling and optimizing the nonlinear, interactive responses characteristic of fungal fermentations (Silva and Cardoso 2024; Yılmaz and Şahan 2020).

The burgeoning global market for natural nutraceuticals, including B vitamins and carotenoids, has intensified the search for sustainable and economically feasible bioproduction strategies (Nazir et al. 2024). In this context, microbial biosynthesis utilizing food waste presents an environmentally sound and cost‐effective alternative (Igreja et al. 2021). For carotenoid production, species such as *N*. *intermedia*, *Talaromyces* spp., *Fusarium fujikuroi*, and various *Penicillium* and *Eurotium* strains are recognized as prolific pigment producers (Gmoser 2021). Simultaneously, diverse fungal species demonstrate specialized biosynthesis of essential B vitamins: Notable producers include *Ashbya gossypii* and *Eremothecium ashbyii* for riboflavin (B2), *Aspergillus niger* for niacin (B3) and riboflavin, and *Cercospora nicotianae* for pyridoxine (B6), underscoring their significant potential for integrated fermentation processes in food and industrial applications (Dikkala et al. 2022).

This manuscript demonstrates three novel contributions, including the first systematic co‐production of six value‐added products from cheese whey using *N*. *intermedia*, specifically mycoprotein, carotenoids, and vitamins B1, B2, B3, and B6. It also presents RSM resolution of contradictory optima with 40 g/L whey for biomass versus 10 g/L for nutraceuticals. The third contribution is the establishment of pellet morphology as a tunable lever that inversely correlates with metabolite production.

This study addresses the critical research gap in developing a systematic optimization strategy for the simultaneous co‐production of mycoprotein, multiple B vitamins (B1, B2, B3, B6), and carotenoids from cheese whey using *N*. *intermedia*, aiming to valorize this byproduct through an integrated bioprocessing platform. The specific objectives are to optimize fungal biomass as high‐quality mycoprotein; enhance the concurrent yield of the spectrum of B1, B2, B3, and B6 vitamins and carotenoids; and systematically identify the optimal cultivation conditions by employing an integrated OFAT and RSM approach to refine key parameters (pH, whey concentration, and inoculum level) for maximizing the multi‐product output of this whey‐based biorefinery.

## 2 Materials and Methods

### 2.1 Whey Characteristics

Whey powder was obtained from Pegah Dairy Company (Isfahan, Iran). This powder is derived from the liquid whey, a byproduct of cheese production, which is dehydrated to form a stable powder. The whey powder was stored at 4°C until use in experiments (Kaya et al. 2025). Physicochemical and chemical composition of whey is presented in Table 1.

**TABLE 1:** Whey characteristics.

Characteristics	Value	Characteristics (mg/kg)	Value
pH	6.5 ± 0.02	Ca	107.2 ± 0.71
Moisture (%)	2.2 ± 0.01	K	120.5 ± 0.61
Total solids (g/kg)	978.0 ± 0.91	Fe	16.6 ± 0.11
Ash (g/kg)	71.54 ± 0.04	Mg	115.1 ± 0.51
Total fat (%)	0.2 ± 0.01		
Crude protein (%)	13.1 ± 0.01		
Total carbohydrate (%)	7.2 ± 0.01		
Lactic acid (%)	1.7 ± 0.01		
Lactose (g/L)	45 ± 0.1		

### 2.2 Fungal Strain

The filamentous fungus *N*. *intermedia* (accession number ATCC 9276) was procured from the Iranian Research Organization for Science and Technology culture collection. The strain was cultivated and maintained on potato dextrose agar (PDA) slants supplemented with 0.5% (w/v) yeast extract. Slants were incubated under light conditions at 30°C for 14 days to promote sporulation. To prepare the inoculum, spores were harvested by adding 20 mL of sterile distilled water to the slant and gently agitating the surface with a sterile loop. The spore concentration was adjusted to 2.5 × 10^6^ spores/mL using a hemocytometer under a light microscope. The standardized suspension was stored at 4°C and used as the inoculum within 1 month. For fermentation experiments, 1 mL of this spore suspension was used to inoculate each Erlenmeyer flask (Gmoser 2021).

### 2.3 Cultivation in Shaking Flask

Fermentations were conducted in 250 mL Erlenmeyer flasks containing 100 mL of the defined whey medium, inoculated with 1% (v/v) of the standardized spore suspension in Section 2.2. All media components were sterilized by autoclaving at 121°C for 15 min prior to use. Cultivations were performed in a shaking incubator at 30°C and 150 rpm for a period of 5 days. The initial pH of each medium was adjusted using 1 M HCl or 1 M NaOH solutions. Following the incubation period, the fungal biomass was harvested by gentle filtration through a fine‐mesh sieve (1 mm^2^ aperture), washed thoroughly with distilled water, and subsequently dried at 35°C in an oven for 24 h or until a constant weight was achieved (Kaya et al. 2025). The dried biomass was then used for subsequent analyses of biomass yield, carotenoid content, and B‐vitamin concentrations by Section 2.6.1.

A 5‐day (120‐h) cultivation was selected as the optimal harvest window, as preliminary time‐course experiments (0–168 h) showed that maximum biomass accumulated at 96–120 h, carotenoids peaked at 120 h before declining due to photo‐oxidation, and B vitamins plateaued between 96 and 144 h, with all target metabolites reaching near‐maximum concentrations (±5% of peak values) at Day 5.

### 2.4 Screening One Factor at a Time Optimization for Biomass, B Vitamins, and Carotenoids Production

The effects of key cultivation parameters on fungal growth and the production of B1, B2, B3, B6 vitamins, and carotenoids were systematically investigated. Eight distinct variables were studied: whey concentration (5, 10, 15, 25, 40, 60, and 75 g/L), initial pH (4.0, 5.5, 7.0, and 9.0), KNO_3_ concentration (0.5, 1.0, and 1.5 g/L), salt supplementation (0.05 g/L each of MgSO_4_, ZnSO_4_, KH_2_PO_4_, and Na_2_MoO_4_), trace element solution (% v/v: 0.5, 1.0, and 1.5), precursors addition (0.05 g/L each of ammonium acetate, potassium acetate, and potassium citrate), glycerol concentration (% v/v: 2, 3, and 4), and pellet‐inducing agents (2 g/L CaCl_2_, 2% v/v glycerol, and 0.3% v/v Tween 80) (Gmoser et al. 2018; Kaya et al. 2025; Nair et al. 2016). The fungal inoculum was prepared as detailed in Section 2.2 and used to inoculate 100 mL of whey medium in 250 mL Erlenmeyer flasks. All cultivations were performed under standard conditions at 30°C and 150 rpm for 5 days. Fungal growth was quantified by measuring the dry biomass weight (Dianatdar et al. 2025). After 5 days, biomass yield, carotenoid content, and B‐vitamin concentrations were determined as described in Section 2.6.1. The statistical significance of each individual factor was assessed via one‐way ANOVA (*p* value <0.05) using SPSS software (version 26, IBM Corp., Armonk, NY, USA). All experiments were performed in triplicate to ensure data reproducibility.

### 2.5 Box–Behnken Experiment Design for Screening of Optimal Factors for Pelletization, Biomass, B Vitamins, and Carotenoids Production

Box–Behnken design (BBD) was selected to avoid extreme factor combinations, ensure uniform prediction variance, and enable reliable quadratic model fitting. In this study, a three‐factor, three‐level BBD was therefore employed. The independent variables and their coded levels (−1, 0, +1) were pH (4, 6, 9), whey concentration (10, 25, 40 g/L), and inoculum level (0.25, 1.0, 1.75% v/v). The BBD ranges were selected on the basis of preliminary OFAT experiments. pH 4–9 was chosen as growth occurred across this range with optimal metabolite production at pH 4 and 7. Whey concentration 10–40 g/L was selected because biomass was detectable above 10 g/L and substrate inhibition began above 40 g/L. Inoculum 0.25%–1.75% was chosen as lower levels caused extended lag phase (>48 h), whereas higher levels reduced specific productivity. On the basis of preliminary OFAT optimization results, a design matrix of 17 experimental runs was generated using Design‐Expert software (Version 12, Stat‐Ease Inc., Minneapolis, MN, USA), as detailed in Table 3. Dry biomass, pellet size, carotenoids, and B vitamins were analyzed after 5 days as described in Section 2.6.1.

### 2.6 Analytical Methods

#### 2.6.1 Vitamins B and Carotenoids of Fungal Biomass

Carotenoids and B vitamins were extracted from 10 mg of manually milled fungal biomass. The sample was first pre‐treated with 50 µL of dimethyl sulfoxide (DMSO) for 15 min. Subsequently, 1 mL of 99% ethanol was added, and the mixture was subjected to sonication for 5 min to enhance extraction efficiency. For initial process optimization, vitamin concentrations were assessed spectrophotometrically according to method of Dianatdar et al. (2025), but all final reported values were obtained via high‐performance liquid chromatography (HPLC) following the method of Hosain et al. (2021).

Total carotenoid content was measured at 469 nm (Gmoser et al. 2017), and all concentrations are expressed per gram of dry fungal biomass (mg/g for vitamins, µg/g for carotenoids).

#### 2.6.2 Analytical Inductively Coupled Plasma (ICP) Metals and Chemical Oxygen Demand (COD) of Whey

COD of the whey medium was monitored using a standard Hach test kit (Hach Company, USA) at two key stages: initial (pre‐cultivation) and post‐cultivation, following the procedure described by Almeida Medeiros et al. (2025). Elemental analysis was performed using ICP optical emission spectrometry (ICP‐OES; Model PQ 9000, Analytik Jena, Germany) in accordance with ASTM‐D1976‐20. For liquid whey samples post‐cultivation, a 10 mL aliquot was digested with a mixture of concentrated nitric acid (HNO_3_, 2 mL) and hydrochloric acid (HCl, 3 mL) for 2 days. For solid ash analysis, 0.05 g of ash whey powder (450°C for 3 h) was digested with a mixture of concentrated HNO_3_ (2 mL) and HCl (6 mL) at 100°C for 1 h until complete decolorization. All digests were filtered through a 0.22 µm membrane prior to ICP‐OES analysis (Elgammal et al. 2019).

#### 2.6.3 HPLC Sugar

The quantification of lactose was performed using HPLC. Chromatographic separation was achieved on an amino (NH_2_) stationary phase column (250 mm × 4.6 mm, 5 µm particle size) maintained at 45°C. The mobile phase consisted of a mixture of acetonitrile and water (70:30, v/v) delivered at an isocratic flow rate of 1.0 mL/min (Association 2005).

#### 2.6.4 CHNS Elemental Analysis

The elemental composition (carbon, hydrogen, nitrogen, and sulfur) of cheese whey powder and *N*. *intermedia* fungal biomass was determined using a Leco‐932 CHNS analyzer (Leco Corporation, St. Joseph, MI, USA) at the Central Laboratory of the University of Isfahan (Isfahan, Iran). Approximately 2–3 mg of each dried, homogenized sample was accurately weighed into tin capsules and combusted at 950°C in a pure oxygen atmosphere (Tiwari and Prajapati 2024).

## 3 Results and Discussion

In this study, the edible filamentous fungus *N*. *intermedia* was evaluated as a biocatalyst for multi‐product valorization of cheese whey, with the objective of converting a major dairy byproduct into a portfolio of value‐added compounds while improving overall process performance. Controlled submerged cultivations were performed and combined with response RSM to quantify how key operating variables influenced fungal growth, pellet morphology, and metabolite formation. Across the investigated experimental space, cheese whey was consistently converted into protein‐rich fungal biomass (mycoprotein) together with co‐produced bioactive/micronutrient fractions, notably carotenoids and B vitamins, and these responses were found to vary systematically with cultivation conditions and morphological characteristics. In parallel, changes in the whey matrix were monitored to assess the extent of substrate conversion and potential co‐benefits for whey management. Overall, the results indicate that *N*. *intermedia* can serve as a robust platform organism for whey‐based fermentation, enabling the simultaneous generation of food‐relevant ingredients and providing a quantitative basis for selecting conditions that balance biomass formation with targeted micronutrient production before the detailed effects and optimization outcomes are presented in the following sections.

### 3.1 One Factor at a Time Optimization

#### 3.1.1 Whey, KNO_3_, and Precursors

**FIGURE 1:**
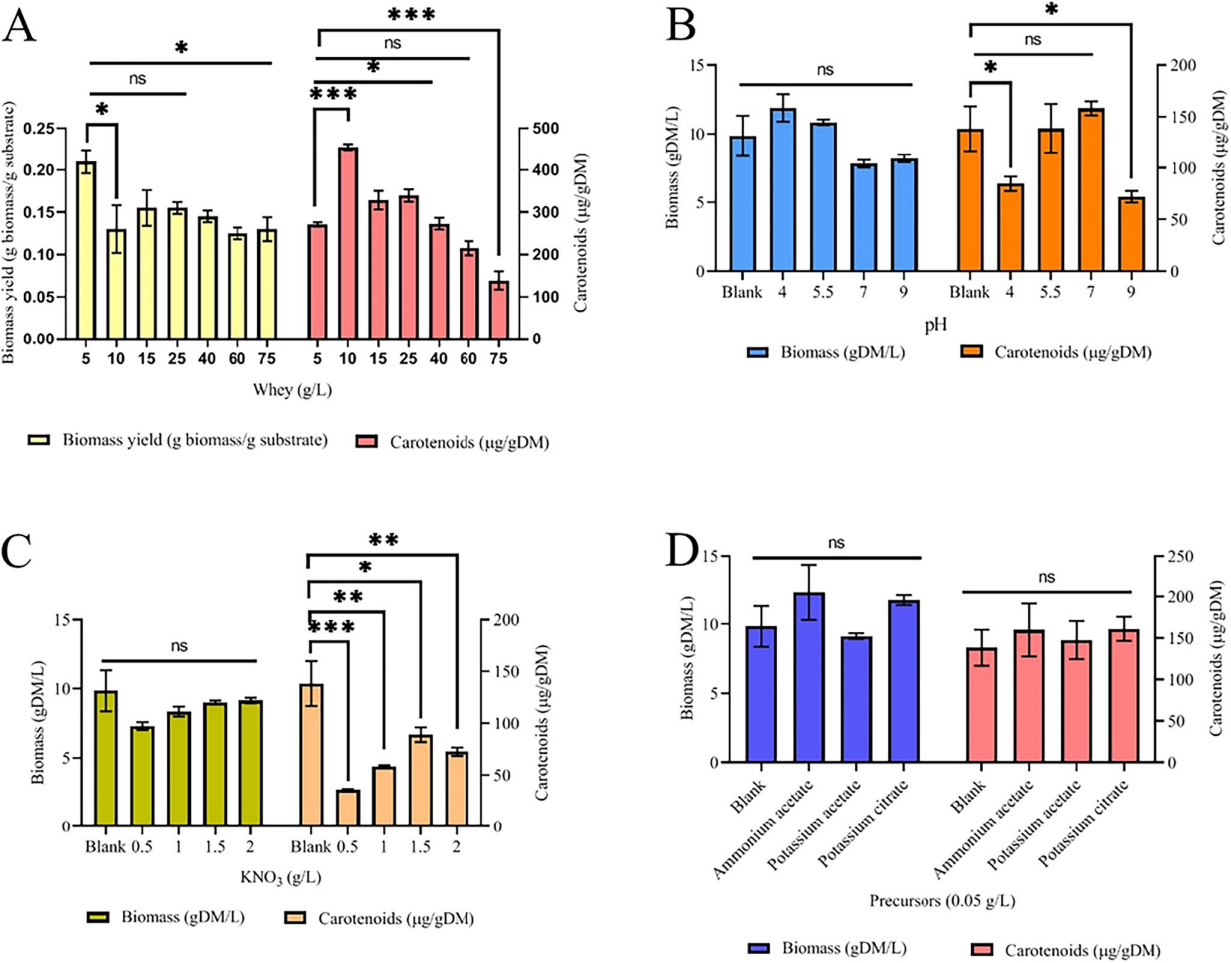
Effect of physiological factors on biomass and carotenoid production by *Neurospora intermedia*. (A) whey concentration (g/L), (B) pH, (C) KNO3 concentration (g/L), (D) precursors.

The effect of whey concentration on the production of biomass, carotenoids, and B vitamins was evaluated (Figure 1, Table 2). Although total fungal biomass increased proportionally with whey concentration (5–75 g/L), the biomass yield per gram of substrate (g biomass/g whey) declined significantly from 0.21 to 0.12, indicating diminishing returns and potential substrate inhibition at higher whey concentrations. Carotenoid biosynthesis exhibited a distinct, sharp optimum at 10 g/L. Concentrations below and above this optimum (5, 25, 15, 40, 60, and 75 g/L) resulted in markedly lower carotenoid yields. This pattern suggests that a moderate nutrient load (10 g/L) creates an ideal physiological state for secondary metabolism, likely by providing sufficient carbon flux without inducing the catabolite repression or osmotic stress that may occur at higher concentrations (Figure 1A and Table 2). On the basis of OFAT results, 25 g/L whey was selected for subsequent experiments as it balanced biomass recovery and maximal carotenoid production.

**TABLE 2:** Impact of physiological factors on B‐vitamin production by *Neurospora intermedia* on the yields of vitamins B1, B2, B3, and B6.

Factors		Vitamin B1 ± SD (*p* value)	Vitamin B2 ± SD (*p* value)	Vitamin B3 ± SD (*p* value)	Vitamin B6 ± SD (*p* value)
Whey (g/L)	5	1.76 ± 0.38	5.55 ± 0.39	0.36 ± 0.01	1.35 ± 0.23
	10	1.73 ± 0.45^N^	5.15 ± 0.01^N^	0.31 ± 0.03^N^	1.12 ± 0.14^N^
	15	2.00 ± 0.52^N^	5.20 ± 1.96^N^	0.34 ± 0.05^N^	1.09 ± 0.36^N^
	25	1.44 ± 0.09^N^	5.41 ± 0.81^N^	0.27 ± 0.03^N^	0.97 ± 0.12^N^
	40	1.34 ± 0.09^S^	4.29 ± 0.18^S^	0.25 ± 0.00^S^	0.95 ± 0.13^S^
	60	1.26 ± 0.27^S^	3.63 ± 0.07^S^	0.22 ± 0.00^S^	0.96 ± 0.34^S^
	75	1.36 ± 0.07^S^	3.30 ± 0.18^S^	0.23 ± 0.01^S^	0.92 ± 0.06^S^
pH	Blank^a^	1.36 ± 0.07	3.30 ± 0.18	0.23 ± 0.01	0.91 ± 0.06
	4	1.63 ± 0.21^N^	4.94 ± 1.11^N^	0.23 ± 0.03^S^	1.09 ± 0.21^N^
	5.5	1.56 ± 0.23^N^	4.82 ± 0.43^N^	0.25 ± 0.03^S^	1.00 ± 0.26^N^
	7	1.32 ± 0.17^N^	4.44 ± 0.28^N^	0.29 ± 0.01^N^	0.96 ± 0.19^N^
	9	1.60 ± 0.39^N^	4.71 ± 0.40^N^	0.26 ± 0.04^S^	1.09 ± 0.18^N^
KNO_3_ (g/L)	Blank^a^	1.36 ± 0.07	3.30 ± 0.18	0.23 ± 0.01	0.91 ± 0.06
	0.5	1.31 ± 0.17^N^	4.74 ± 0.3^S^	0.26 ± 0.01^S^	0.87 ± 0.04^N^
	1	1.23 ± 0.06^N^	4.42 ± 0.54^S^	0.27 ± 0.03^S^	0.83 ± 0.03^N^
	1.5	1.15 ± 0.18^N^	4.35 ± 0.12^S^	0.26 ± 0.04^S^	0.86 ± 0.04^N^
	2	1.33 ± 0.06^N^	4.39 ± 0.28^N^	0.2 ± 0^N^	0.79 ± 0.08^S^
Precursor	Blank^b^	1.36 ± 0.17	4.74 ± 0.3	0.26 ± 0.01	0.87 ± 0.04
	Ammonium acetate	1.95 ± 0.55^N^	5.45 ± 0.09^N^	0.33 ± 0.01^N^	0.91 ± 0.04^N^
	Potassium acetate	1.58 ± 0.01^N^	5.17 ± 0.2^N^	0.27 ± 0.01^N^	1.01 ± 0.32^N^
	Potassium citrate	1.57 ± 0.29^N^	6.56 ± 0.85^S^	0.3 ± 0.03^N^	0.95 ± 0.06^N^

Abbreviations: N, nonsignificant; S, significant.

^a^70 g/L whey, pH 7.

^b^70 g/L whey, pH 7, and 0.5 g/L KNO_3_.

The biomass yield of 0.21 g biomass/g substrate obtained at 5 g/L whey aligns closely with the 0.21 g/g reported for *N. intermedia* by Kaya et al. (2024) and is higher than the 0.12 g/g from an earlier study (Mahboubi et al. 2017a), reinforcing the reproducibility of such fermentations. Importantly, our data reveal that this efficiency is not static but is highly dependent on initial substrate concentration, declining significantly from 0.21 to 0.12 g/g as whey concentration increased from 5 to 75 g/L. This pattern of diminishing returns and potential substrate inhibition at higher loads helps explain the yield ranges reported in the literature and underscores that maximizing absolute biomass (Kaya et al. 2024) involves optimizing not only scale and aeration but also the fundamental substrate concentration to avoid inhibitory effects. Furthermore, the distinct, sharp optimum for carotenoid production at 10 g/L, decoupled from the linear biomass increase, highlights the critical physiological trade‐off between primary growth and secondary metabolism in these systems.

The influence of nitrogen source and metabolic precursors was highly specific. Supplementation with KNO_3_ (0.5–1.5 g/L) and potassium citrate did not enhance biomass or carotenoid yields, suggesting that growth and pigment synthesis were not nitrogen‐ or precursor‐limited under the tested conditions. However, vitamin B2 and B3 levels increased under selected conditions (Figure 1C,D; Table 2). Supplementation with KNO_3_ (0.5–1.5 g/L) did not enhance biomass or carotenoid yields (*p* > 0.05), confirming that the native nitrogen content of whey (crude protein 13.3%) was sufficient to meet *N*. *intermedia's* metabolic demands. The addition of 1.5 g/L KNO_3_ would increase total solids by only 0.15%, making it technically feasible but economically unnecessary. This finding simplifies medium formulation and supports the economic viability of using whey as a standalone cultivation substrate without external nitrogen fortification. One plausible explanation is that nitrate assimilation is reductant‐demanding and can increase cellular demand for redox cofactors (FAD/FMN and NAD(P)), which may elevate the biosynthetic flux toward their vitamin precursors. The enhancement observed with potassium citrate may additionally reflect improved provision of carbon skeletons into the riboflavin pathway or altered intracellular redox balance.

Baseline vitamin levels of whey were quantified at approximately 655 mg/kg (16.4 mg/L in 25 g/L whey medium). Net vitamin biosynthesis by *N*. *intermedia* reached 25.82 mg per liter (42.22 mg in harvested biomass minus 16.4 mg initial), representing a 2.6‐fold enrichment and confirming active B‐vitamin production rather than mere recovery from the whey substrate.

#### 3.1.2 Salts

Supplementation of the whey medium with various salts (MgSO_4_, ZnSO_4_, KH_2_PO_4_, Na_2_MoO_4_), different concentrations of zinc and molybdenum, or trace element solutions (0.5%–1.5% v/v) did not yield significant increases in the production of biomass, carotenoids, or B vitamins (Figure S1A–C, Table S1). This indicates that the intrinsic mineral composition of the whey substrate was sufficient to meet the nutritional demands of *N*. *intermedia* under the tested conditions. The lack of response to supplementation aligns with the established critical ion requirements for this fungus (specifically Ca^2^^+^ (0.8 g/L), Mg^2^^+^ (0.17 g/L), and Zn^2^^+^ (0.002 g/L)), which are naturally present in appreciable quantities within the whey matrix (Dianatdar et al. 2025; see Table 1). Consequently, no external mineral fortification was necessary, simplifying the medium formulation and supporting the economic viability of using whey as a standalone cultivation substrate.

In contrast to Dianatdar et al. (2025), who reported beneficial effects of individual Zn or Mo supplementation on carotenoid production in *N. intermedia*, our study specifically tested the combined addition of Zn + Mo (0.05 g/L each), which unexpectedly reduced carotenoid yield (Figure S1C). This antagonistic effect likely arises from competitive inhibition between Zn^2^^+^ and MoO_4_^2^^−^ for shared membrane transporters (e.g., sulfate permease family), or from supraoptimal metal concentrations triggering metal‐induced oxidative stress and feedback suppression of the carotenoid biosynthesis pathway (Sánchez et al. 2025). Notably, individual metals were not tested separately in this study, as the OFAT screening prioritized evaluation of the combination. Future dose–response studies across a broader concentration range are warranted to determine whether lower combined doses might recapitulate the beneficial effects observed with individual supplementation.

#### 3.1.3 Pellet‐Inducing Agents

Pellet‐inducing agents, specifically 2 g/L CaCl_2_, glycerol (2, 3, and 4% v/v), and 0.3% v/v Tween 80 had no significant effect on pellet formation, biomass yield, carotenoid, or B‐vitamin production (Figure S1D,E and Table S1). Nair et al. (2016) reported that additive agents (glycerol and CaCl_2_) and trace metals did not influence the pelletization of *N*. *intermedia*; they identified pH (2–3), inoculum level, and glucose concentration as significant factors. Building on this, we investigated pH, inoculum size, and whey concentration, which served as the combined carbon and nitrogen source, as critical parameters for pelletization using a BBD as detailed in Section 3.2.7.

Under optimal conditions OFAT (25 g/L whey, pH 7, 1 g/L potassium citrate, 30°C, 150 rpm), the B‐vitamin content of *N*. *intermedia* biomass was analyzed using both UV–Vis spectrophotometry and HPLC. The concentrations obtained by UV–Vis were 1.31 mg/g for vitamin B1, 6.05 mg/g for B2, 0.28 mg/g for B3, and 0.89 mg/g for B6, which closely matched the HPLC validation results (Pearson's *r* > 0.94), confirming the reliability of UV–Vis quantification for screening purposes (Figure S2).

### 3.2 Experiment Design and Determination of Optimal Conditions by Box–Behnken Method

**FIGURE 2:**
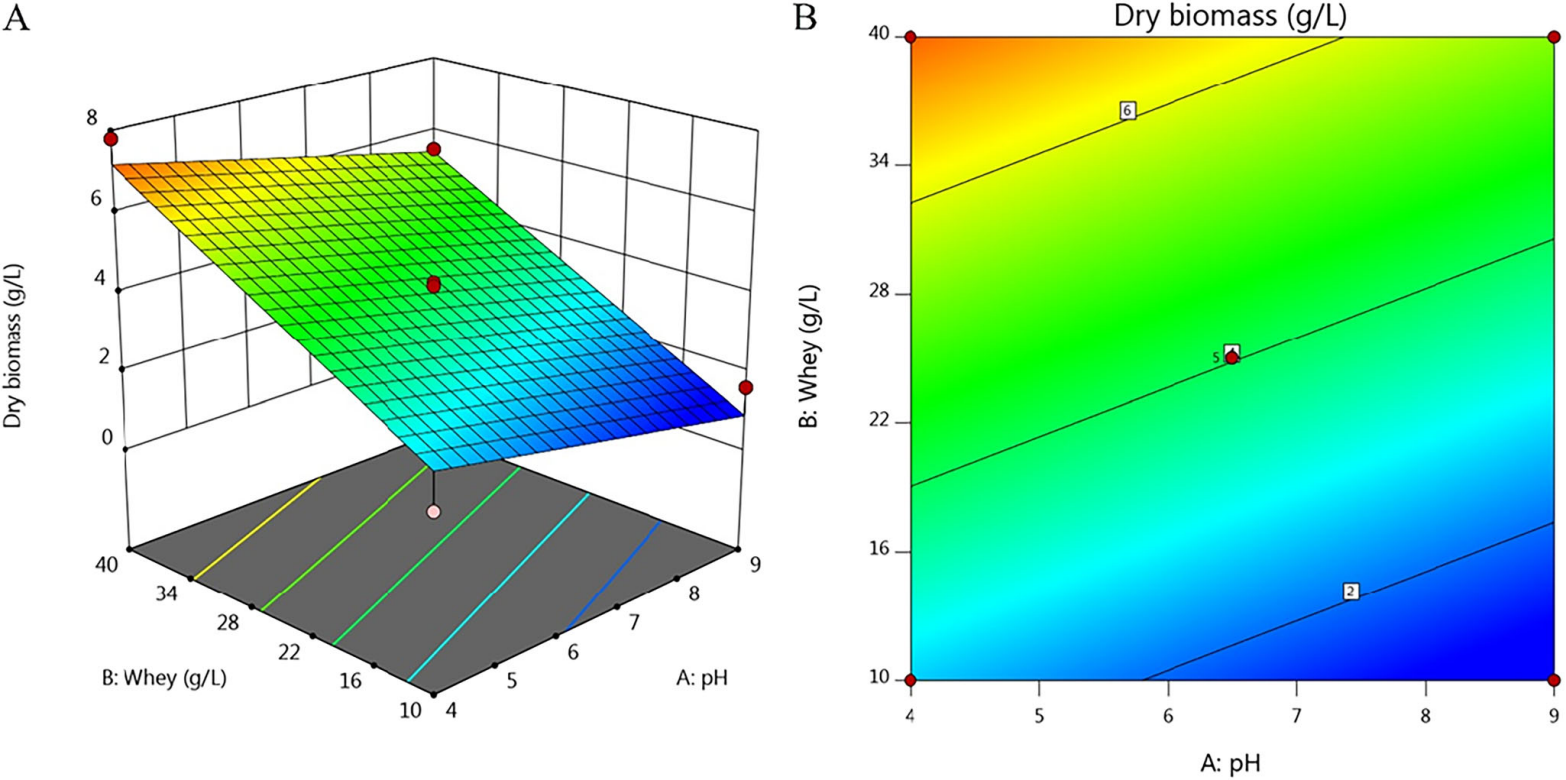
Response surface analysis illustrating the interactive effects of process variables on dried biomass production (g DM/L) by *Neurospora intermedia* in whey medium at 30°C and 150 rpm. (A) the 3D plots whey (g/L) and pH, (B) the contour plots whey (g/L) and pH.

Initial optimization using the OFAT method established a baseline condition conducive to the concurrent production of biomass, carotenoids, and B vitamins. At 25 g/L whey concentration and pH 7, this condition resulted in a dried biomass yield of 3.85 g/L. Under these conditions, carotenoid and vitamin yields were substantial: 169.22 µg/g for carotenoids, and 1.62, 4.44, 0.27, and 0.94 mg/g for vitamins B1, B2, B3, and B6, respectively (Table 2). To elucidate factor interactions and further optimize the process, RSM was employed. The RSM model revealed a significant interaction between pH and whey concentration. Notably, the model identified a distinct optimum for dried biomass production, predicting and subsequently validating a yield of 7.8 g/L under more acidic conditions (pH 4) and a higher substrate load (40 g/L whey) with a 1% (v/v) inoculum (Run 11, Table 3) (Figure 2). The subsequent RSM optimization identified a condition (Run 12: pH 4, 10 g/L whey, 1% v/v inoculum) that minimized pellet size, yielding a compact pellet of only 2 mm. This morphology was not observed in any of the OFAT experiments.

**TABLE 3:** Box–Behnken experimental design matrix with independent variables (*A*: pH, *B*: whey, *C*: inoculum level) and measured responses.

Variables				Responses						
Run	*A*: pH	*B*: Whey (g/L)	*C*: Inoculum level (%v/v)	Dry biomass (g/L)	Carotenoids (µg/g)	Vitamin B1 (mg/g)	Vitamin B2 (mg/g)	Vitamin B3 (mg/g)	Vitamin B6 (mg/g)	Size pellet (mm)
1	6.5	40	1.75	5.7	289.84	1.01	4.71	0.21	0.75	0
2	4	25	1.75	5.6	411.56	1.18	6.40	0.37	0.93	2.1
3	9	25	0.25	3.2	135.58	1.19	6.43	0.33	1.20	5.1
4	6.5	25	1	4	333.12	1.29	5.22	0.25	0.97	11.5
5	6.5	25	1	4.3	307.86	1.28	5.33	0.26	0.93	12.2
6	6.5	10	0.25	1.6	366.88	1.39	7.01	0.39	1.21	4.1
7	9	25	1.75	3.6	164.42	1.23	6.28	0.32	0.87	0
8	9	10	1	1.6	205.04	1.43	7.26	0.39	1.02	4.1
9	6.5	10	1.75	1.3	464.84	1.39	6.95	0.38	1.20	0
10	6.5	25	1	3.7	322.64	1.32	5.25	0.25	1.06	11.3
11	4	40	1	7.8	249.76	1.07	4.54	0.31	0.99	3.6
12	4	10	1	1.7	499.92	1.46	7.17	0.41	1.20	2
13	6.5	25	1	3.5	339.42	1.29	5.39	0.25	1.09	10.5
14	4	25	0.25	5.8	199.84	1.24	6.00	0.31	1.08	3.1
15	6.5	40	0.25	5.4	265.50	0.97	4.84	0.22	1.16	0
16	9	40	1	5.5	127.78	1.11	4.72	0.21	1.09	0
17	6.5	25	1	4.2	318.50	1.29	5.61	0.27	1.02	10.9

#### 3.2.1 Biomass

Statistical validation confirmed the robustness of a linear model for predicting dried biomass production in a whey‐based bioprocess. Analysis of variance demonstrated the model's high significance with an *F* value of 39.92 and a *p* value below 0.0001. The model exhibited excellent explanatory power, evidenced by an *R* ^2^ value of 0.9021, indicating it explains over 90% of the variability in biomass production. Its reliability was further supported by the close agreement between adjusted *R* ^2^ (0.8795) and predicted *R* ^2^ (0.8040). A nonsignificant lack of fit (*p* > 0.05) confirmed the model's strong predictive capability without overfitting the data. Analysis of the regression coefficients revealed that whey concentration (*B*) was the most influential factor, exerting a strong positive effect (+2.28) on biomass yield. In contrast, pH (*A*) showed a substantial negative effect (−0.8750), whereas inoculum size (*C*) had a negligible impact (+0.0250). These relationships were captured in the following equation:

(1) Drybiomass=+4.03−0.8750A+2.28B+0.0250C

RSM further uncovered a significant interactive effect between pH and whey concentration. This interaction was visualized through elliptical contours and pronounced curvature in both 2D and 3D plots. The analysis pinpointed the optimal conditions for maximizing biomass yield at 40 g/L whey and pH 4.0. This specific optimum represents a critical balance where the whey provides sufficient carbon, whereas the acidic pH enhances nutrient solubility and uptake. Below approximately 30 g/L whey, growth is limited by carbon availability, resulting in efficient substrate use but low total biomass. Above 40 g/L, although absolute biomass might increase, the yield efficiency drops sharply due to ionic stress from supraoptimal cation levels. Thus, the RSM‐derived optimum strategically positions maximum productivity at the transition point before severe ionic inhibition outweighs the benefits of additional carbon.

The optimal biomass production of *N*. *intermedia* at pH 4.0 aligns with the well‐documented capacity of filamentous fungi to maintain intracellular pH homeostasis even under acidic external conditions. As demonstrated by Hesse et al. (2002) in *A. niger*, fungi can sustain a constant cytoplasmic pH (pHcyt) of around 7.6 across a wide range of extracellular pH (pHex) values. The acidic environment likely enhances the solubility of nutrients in the whey, and the fungus can leverage its robust pH homeostasis mechanisms to thrive.

#### 3.2.2 Carotenoids

**FIGURE 3:**
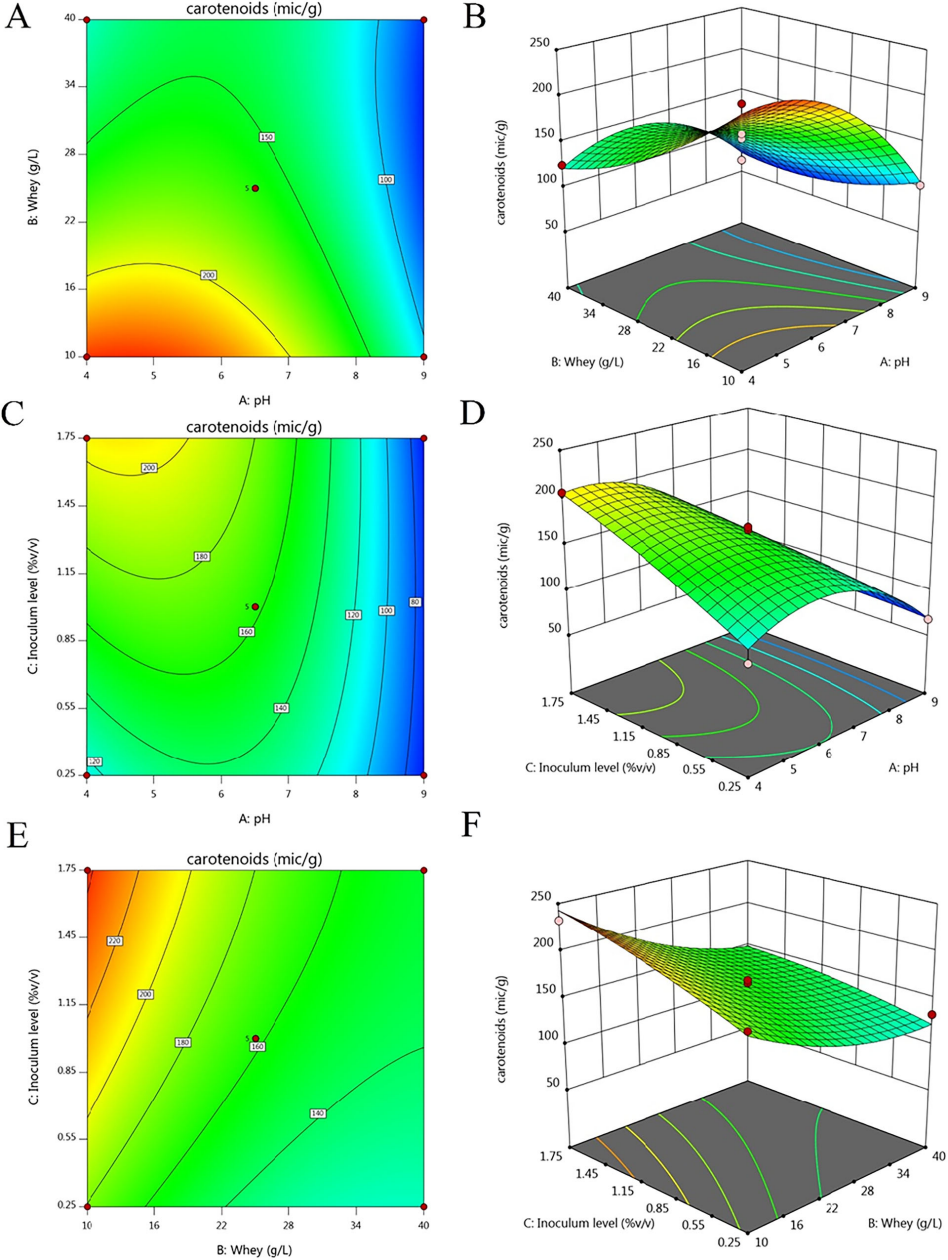
Response surface analysis illustrating the interactive effects of process variables on carotenoid production (µg/g) by *Neurospora intermedia* in whey medium at 30°C and 150 rpm. (A) the contour plots whey (g/L) and pH, (B) the 3D plots whey (g/L) and pH, (C) the contour plots inoculum level (%) and pH, (D) the 3D plots inoculum level (%) and pH, (E) the contour plots inoculum leve (%) and whey (g/L), (F) the 3D plots inoculum level (%) and whey (g/L).

A highly significant quadratic response surface model (*F* value = 34.51, *p* < 0.0001) was developed to describe carotenoid production by *N*. *intermedia*, demonstrating exceptional explanatory power with an *R* ^2^ of 0.9780 and an adjusted *R* ^2^ of 0.9496, whereas a high adequate precision ratio of 18.88 confirmed its reliability for prediction (Figure 3). The model revealed that pH (*A*) and whey concentration (*B*) were the dominant factors with strong linear effects (*p* < 0.0001), whereas significant quadratic terms for both variables (*A* ^2^, *p* = 0.0002; *B* ^2^, *p* = 0.0276) indicated that carotenoid yield reaches distinct maxima at intermediate levels rather than following a linear relationship. Additionally, significant two‐factor interactions between pH and whey concentration (*AB*, *p* = 0.0089) and between pH and inoculum level (*AC*, *p* = 0.0068) demonstrated that the effect of each variable depends on the levels of others, justifying the response surface approach over simpler optimization methods (Table S3). The complete regression equation (Equation 2) effectively captures these complex, nonlinear relationships and provides a reliable framework for optimizing carotenoid production:

(2) $$\begin{array}{ccc} \mathrm{Carotenoids} & = & +\;162.15\;-\;45.52A\;-\;37.74B+22.68C\\ & & +\;21.61AB\;-\;22.86AC\;-\;9.20BC\\ & & \;-\;43.15A^{2}+16.31B^{2}\;-\;5.08C^{2} \end{array}$$

RSM modeling identified optimal carotenoid biosynthesis by *N*. *intermedia* under low pH (4), low whey concentration (10 g/L), and high inoculum levels, which collectively induce a controlled physiological stress state. These conditions, carbon limitation from low substrate, acidic environmental stress, and rapid population establishment from high inoculum, synergistically redirect metabolic flux from primary growth toward secondary metabolism, specifically enhancing production of protective carotenoid pigments. The significant antagonistic interaction between pH and inoculum level in the statistical model demonstrates how cell density modulates the stress response, with high initial biomass accelerating synchronized nutrient depletion. This mechanistic framework, where rapid carbon exhaustion under acidic, growth‐limiting conditions creates an optimal physiological niche for secondary metabolite synthesis, aligns with established principles of fungal metabolism and provides a transferable strategy for optimizing bioactive compound production in filamentous fungi. Gmoser (2021) reported that the optimal conditions for carotenoid production by *N*. *intermedia*, low pH, low substrate concentration, and high inoculum align with the established view of these pigments as protective secondary metabolites synthesized under environmental stress.

#### 3.2.3 Vitamin B1

Vitamin B1 production by *N*. *intermedia* was precisely modeled using a statistically robust quadratic equation (Equation 3), validated by a highly significant *F* value (105.56, *p* < 0.0001), an exceptional fit to experimental data (*R* ^2^ = 0.9927), and a nonsignificant lack of fit (*p* = 0.2565), with a high predicted *R* ^2^ (0.9252) confirming excellent predictive capability. Analysis revealed whey concentration (*B*) as the dominant factor exerting a strong negative linear effect (coefficient −3.73, *p* < 0.0001), whereas pH (*A*) and inoculum level (*C*) showed no significant linear influence. The model captured complex system behavior through significant quadratic terms for whey concentration (*B* ^2^, *p* = 0.0212) and inoculum level (*C* ^2^, *p* < 0.0001), indicating that B1 production reaches optimal levels at specific intermediate values of these factors, whereas all two‐factor interactions (*AB*, *AC*, *BC*) displayed positive synergistic relationships where combined effects enhanced yield beyond individual contributions (Table S4). The complete Equation (3) effectively captures these relationships, establishing whey concentration as the primary limiting factor and demonstrating the value of RSM for optimizing vitamin B1 biosynthesis in this system.

(3) $$\begin{array}{ccc} & & \mathrm{Vitamin}\;B1=+25.94+0.0104A\;-\;3.73B+0.0276C+0.3510AB\\ & & +\;0.4758AC+0.2134BC\;-\;0.1045A^{2}\;-\;0.5236B^{2}\;-\;1.58C^{2} \end{array}$$

**FIGURE 4:**
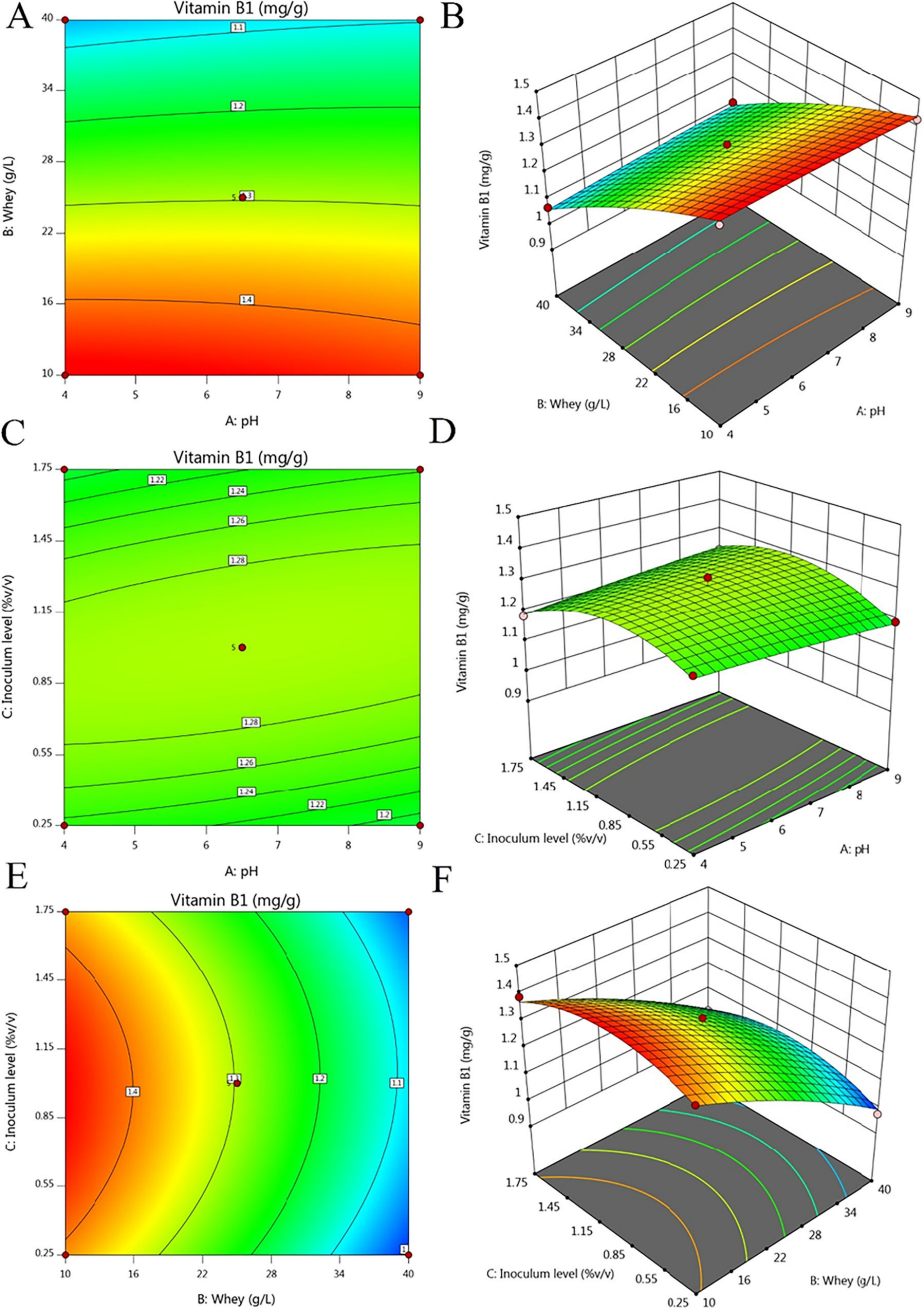
Response surface analysis illustrating the interactive effects of process variables on B1 vitamin (mg/g) production by *Neurospora intermedia* in whey medium at 30◦C and 150 rpm. (A) the contour plots whey (g/L) and pH, (B) the 3D plots whey (g/L) and pH, (C) the contour plots inoculum level (%v/v) and pH, (D) the 3D plots inoculum level (%v/v) and pH, E) the contour plots inoculum level (%v/v) and whey (g/L), F) the 3D plots inoculum level (%v/v) and whey (g/L).

The enhanced production of vitamin B1 by *N*. *intermedia* under optimized conditions is a direct consequence of its function as a growth‐coupled primary metabolite, with RSM (Figure 4) providing a statistical validation of this physiological principle. During the trophophase, elevated biosynthetic flux is necessitated by the high demand for thiamine pyrophosphate, an indispensable coenzyme for key metabolic nodes, including transketolase in the pentose phosphate pathway and the dehydrogenase complexes of the TCA cycle, that drive respiratory growth and anabolism (Palmieri et al. 2022). Consequently, process parameters that promote rapid and sustained mycelial development inherently stimulate thiamine synthesis, a concept graphically confirmed by the empirical models. The valley‐shaped response for whey concentration‐pH (Plots *A* and *B*) visualizes the dominant negative constraint of high substrate levels, whereas the saddle‐shaped surface for inoculum–pH (Plots *C* and D) resolves a significant interactive effect pinpointing an optimal inoculum size. Crucially, the pronounced ridge in the inoculum–whey interaction (Plots *E* and *F*) demonstrates that strategic modulation of the inoculum can partially mitigate substrate inhibition.

#### 3.2.4 Vitamin B2

The quadratic model for vitamin B2 (riboflavin) production by *N*. *intermedia* demonstrated exceptional explanatory power, accounting for 98.58% of yield variability (*R* ^2^ = 0.9858), with robust statistical validation including a nonsignificant lack of fit (*p* = 0.3829), high adequate precision ratio (19.85), and strong predictive capability (predicted *R* ^2^ = 0.8752). Factor analysis revealed whey concentration (*B*) as the overwhelmingly dominant linear factor with a strong negative effect (coefficient −23.95, *p* < 0.0001), whereas pH (*A*) and inoculum level (*C*) showed no significant linear influence. The model captured unique system behavior through significant positive quadratic terms for all three factors (*A* ^2^, *p* = 0.0006; *B* ^2^, *p* = 0.0011; *C* ^2^, *p* = 0.0011), indicating a convex response surface where riboflavin yield is minimized near the center of the experimental space and increases toward factor boundaries (Table S5). Notably, no significant interaction effects were detected between variables, except for a negative pH–inoculum interaction (*AC* = −2.75), suggesting particularly unfavorable combinations. Equation (4) captures the unique convex response profile, establishing whey concentration as the primary inhibitory factor and providing a reliable framework for optimizing riboflavin production at factor boundaries rather than central values:

(4) $$\begin{array}{ccc} & & \mathrm{Vitamin}\;B2=+107.25+1.49A\;-\;23.95B+0.1471C+0.4641AB\\ & & \;-\;2.75AC\;-\;0.3493BC+9.55A^{2}+1.65B^{2}+8.71C^{2} \end{array}$$

**FIGURE 5:**
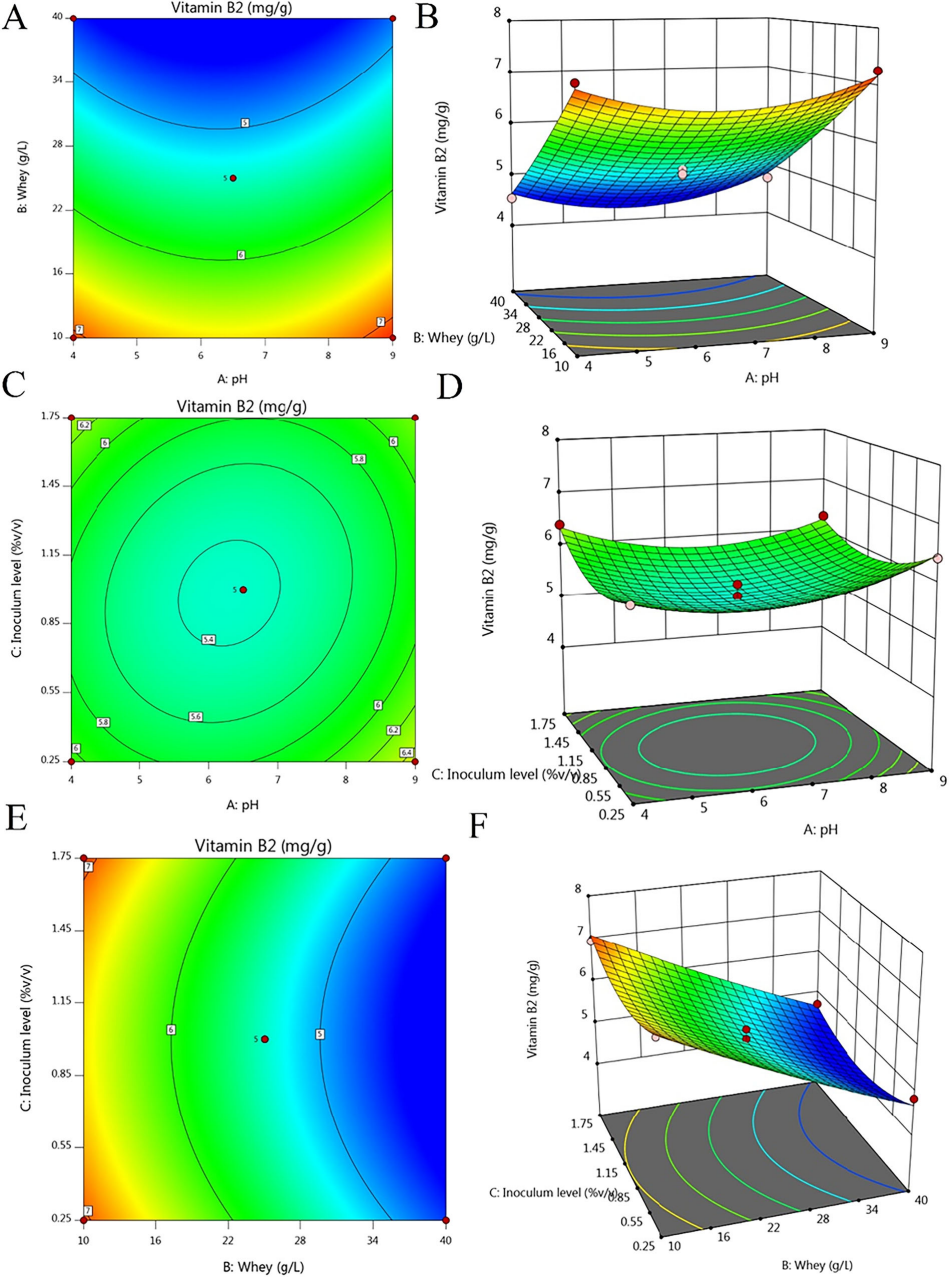
Response surface analysis illustrating the interactive effects of process variables on B2 vitamin (mg/g) production by *Neurospora intermedia* in whey medium at 30◦C and 150 rpm. (A) the contour plots whey (g/L) and pH, (B) the 3D plots whey (g/L) and pH, (C) the contour plots inoculum level (%v/v) and pH, (D) the 3D plots inoculum level (%v/v) and pH, E) the contour plots inoculum level (%v/v) and whey (g/L), F) the 3D plots inoculum level (%v/v) and whey (g/L).

The production of vitamin B2 (riboflavin) presents a notable exception to the general trend of metabolite suppression under high‐nutrient conditions. Although riboflavin yield was also severely inhibited by high whey concentration (Figure 5A,B), its response to pH and inoculum level was uniquely non‐intuitive. The convex, bowl‐shaped response surface (Figure 5C,D) indicates that yield was minimized at central, moderate conditions and increased toward the experimental boundaries. This suggests a regulatory mechanism distinct from catabolite repression. Riboflavin biosynthesis in *N*. *intermedia* may act as a stress‐responsive metabolic shunt. Under the extreme conditions represented by the factor boundaries (e.g., very low or high pH), the filamentous fungi upregulate the riboflavin pathway from GTP to regenerate oxidized cofactors (FAD/FMN) or to counteract oxidative stress (Palmieri et al. 2022). The strong negative linear effect of whey concentration (Figure 5A,B,E,F) remains consistent with classic precursor drainage, where abundant carbon and nitrogen sources divert resources away from specialized cofactor synthesis. Thus, riboflavin production appears to be governed by a dual constraint: it is repressed by nutrient sufficiency yet stimulated by environmental extremity, resulting in its optimal production at the intersection of low whey concentration and noncentral pH/inoculum levels.

#### 3.2.5 Vitamin B3

The quadratic model for vitamin B3 (niacin) production by *N*. *intermedia* was statistically significant (*p* = 0.0002) and demonstrated a good fit to experimental data, explaining 96.63% of variability (*R* ^2^ = 0.9663), with whey concentration (*B*) identified as the most influential factor exhibiting a highly significant negative linear effect (*p* < 0.0001), followed by pH (*A*) with a significant negative linear influence (*p* = 0.0189). The model revealed convex, nonlinear relationships through significant positive quadratic terms for pH (*A* ^2^, *p* = 0.0007) and inoculum level (*C* ^2^, *p* = 0.0413), indicating that niacin yield reaches a minimum near the center of the experimental space and increases toward factor boundaries, whereas all two‐factor interactions (*AB*, *AC*, *BC*) were negative, suggesting antagonistic relationships between variables. However, a significant lack of fit (*p* = 0.0345) and a substantial discrepancy between adjusted *R* ^2^ (0.9230) and the relatively low predicted *R* ^2^ (0.5285) indicate that despite excellent data fitting, the model's predictive capability for new observations is limited, suggesting possible overfitting or the influence of additional unaccounted factors (Table S6). The complete Equation (5) precisely captures these relationships, with all positive quadratic terms confirming the convex response surface where niacin production is optimized at the boundaries rather than central values of the experimental design:

(5) $$\begin{array}{ccc} & & \mathrm{Vitamin}\;B3=+2.56\;-\;0.2035A\;-\;0.7780B+0.0324C\\ & & \;-\;0.2148AB\;-\;0.1693AC\;-\;0.0104BC+0.5292A^{2}+0.2042B^{2}\\ & & +\;0.2303C^{2} \end{array}$$

**FIGURE 6:**
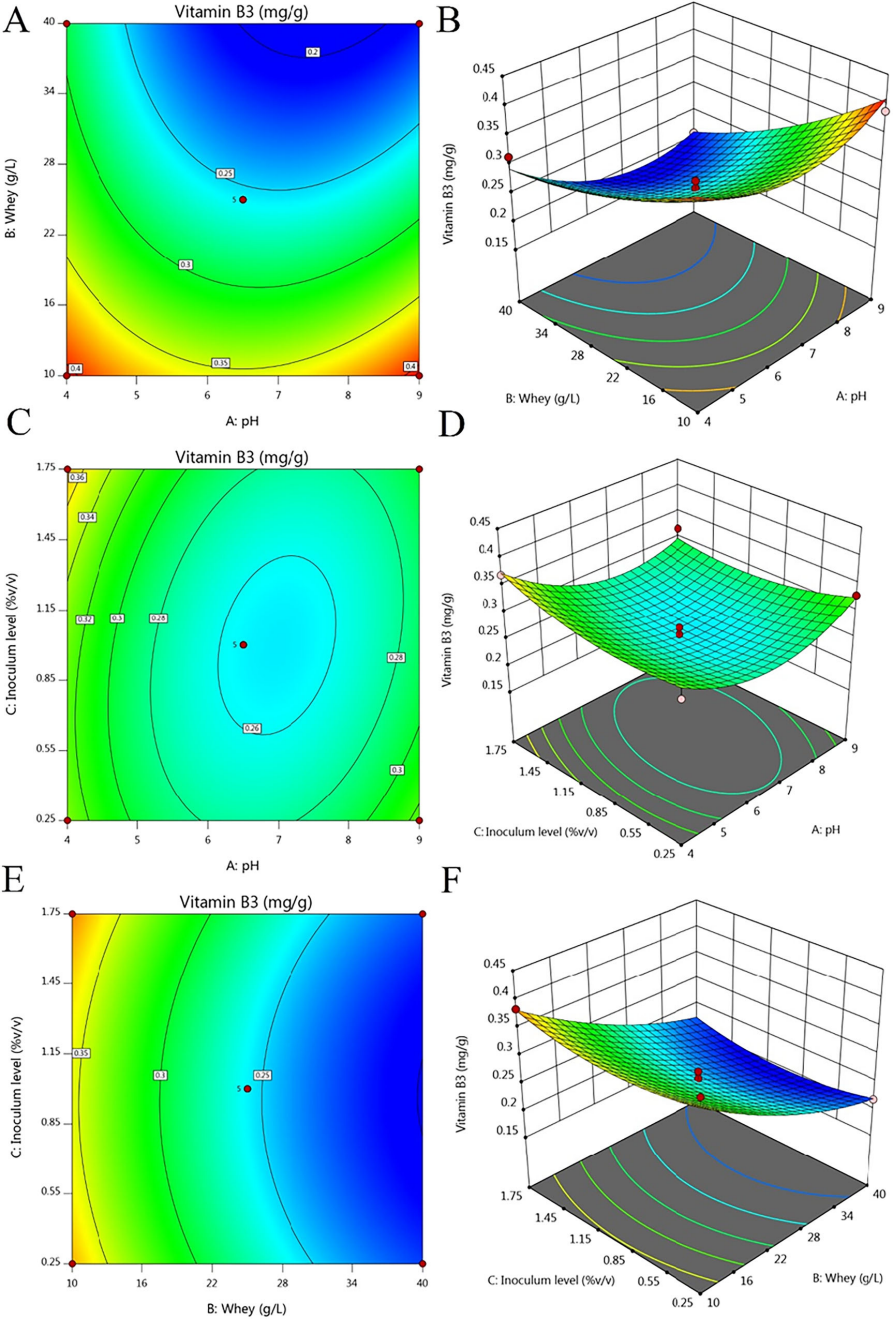
Response surface analysis illustrating the interactive effects of process variables on B3 vitamin (mg/g) production by *Neurospora intermedia* in whey medium at 30◦C and 150 rpm. (A) the contour plots whey (g/L) and pH, (B) the 3D plots whey (g/L) and pH, (C) the contour plots inoculum level (%v/v) and pH, (D) the 3D plots inoculum level (%v/v) and pH, E) the contour plots inoculum level (%v/v) and whey (g/L), F) the 3D plots inoculum level (%v/v) and whey (g/L).

The production of vitamin B3 (niacin), a key NAD(P) precursor, exhibits a response distinct from other metabolites, as visualized in Figure 6. The pronounced valley‐shaped surface for the whey–pH interaction (Plots *A* and *B*) aligns with the classical model of carbon and nitrogen catabolite repression; high concentrations of lactose and amino acids suppress de novo niacin biosynthesis, diverting precursors like aspartate into biomass. More remarkably, the bowl‐shaped valley for the inoculum–pH interaction (Plots *C* and *D*) reveals that niacin yield is minimized under central, balanced conditions. This suggests niacin synthesis in *N*. *intermedia* functions not as a standard secondary metabolite but as a homeostatic redox balancer. Under the metabolic stability of optimal pH and inoculum, NAD(P) pools are likely balanced, and biosynthesis is downregulated. Conversely, at the experimental boundaries, where pH or cell density extremes create metabolic or oxidative stress, the fungus upregulates niacin production to regenerate crucial redox cofactors. Thus, optimal B3 yield is achieved at the intersection of low whey concentration (to avoid nutrient repression) and noncentral pH/inoculum levels (to trigger the homeostatic response), a strategy reflecting its fundamental role in cellular redox metabolism.

Niacin synthesis is minimized under balanced, central conditions but upregulated at environmental extremes to maintain homeostasis. This reflects the essential role of these dinucleotides in mediating cellular redox balance (Xiao et al. 2018).

#### 3.2.6 Vitamin B6

Vitamin B6 production by *N*. *intermedia* was modeled using a two‐factor interaction (2FI) approach, with analysis of variance confirming statistical significance (*F* value = 6.96, *p* = 0.0040) and explaining 80.68% of the observed variance (*R* ^2^ = 0.8068), whereas a nonsignificant lack of fit (*p* = 0.3577) supported model adequacy (Table S7). Factor analysis identified inoculum level (*C*) as the most influential factor, exerting the strongest negative effect on yield (coefficient −2.22, *p* = 0.0014), followed by whey concentration (*B*) with a significant negative linear influence (coefficient −1.63, *p* = 0.0095), whereas pH (*A*) showed no significant linear effect (*p* = 0.9235). A significant interaction between whey and inoculum level (*BC*, *p* = 0.0212) was captured by a substantial negative coefficient (−1.97), indicating a strongly antagonistic effect when both factors are at high levels. The complete Equation (6) quantifies these relationships, though the model is more reliable for identifying influential factors and their interactions than for precise quantitative predictions outside the experimental data, as suggested by its limited predictive capability:

(6) $$\begin{array}{ccc} \mathrm{Vitamin}\;B6 & = & +20.91\;-\;0.0502A\;-\;1.63B\;-\;2.22C+1.37AB\\ & & \;-\;0.8806AC\;-\;1.97BC \end{array}$$

**FIGURE 7:**
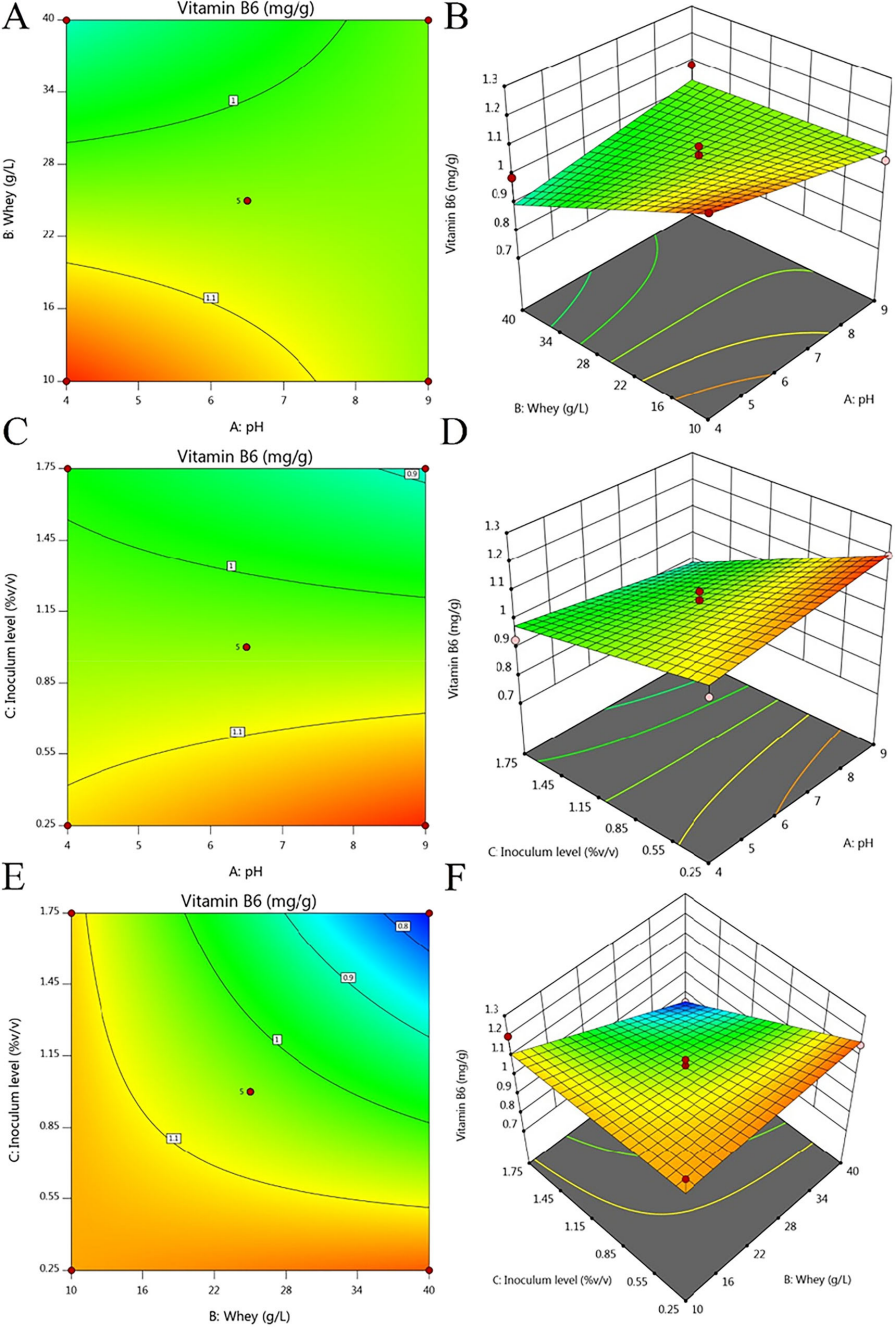
Response surface analysis illustrating the interactive effects of process variables on B6 vitamin (mg/g) production by *Neurospora intermedia* in whey medium at 30◦C and 150 rpm. (A) the contour plots whey (g/L) and pH, (B) the 3D plots whey (g/L) and pH, (C) the contour plots inoculum level (%v/v) and pH, (D) the 3D plots inoculum level (%v/v) and pH, E) the contour plots inoculum level (%v/v) and whey (g/L), F) the 3D plots inoculum level (%v/v) and whey (g/L).

The production of vitamin B6 (pyridoxine) exemplifies the fundamental metabolic trade‐off between biomass growth and cofactor biosynthesis, governed by catabolite repression and precursor drainage. This is clearly visualized in the response surface plots (Figure 7). The uniformly sloping plane of the whey–pH interaction (Plots *A* and *B*) graphically represents the dominant repressive effect of high whey concentration; abundant lactose and amino acids suppress the de novo B6 pathway, diverting key precursors like glycolytic intermediates and glutamate toward growth. The unique saddle‐shaped surface of the whey–inoculum interaction (Plots *E* and *F*) provides deeper mechanistic insight. This shape, directly corresponding to the significant negative *BC* interaction term, reveals that the repressive effect of whey is synergistically amplified by high inoculum levels. Biochemically, a large, dense fungal population on a nutrient‐rich medium maximizes the cellular demand for anabolic precursors, catastrophically starving the B6 biosynthesis route (Iosue et al. 2016). Consequently, the model pinpoints the only favorable operational region: low whey concentration coupled with a moderate inoculum. Under these conditions, precursor drain is minimized, allowing measurable metabolic flux into pyridoxine synthesis. The ridge‐like inoculum–pH structure (Plots *C* and *D*) further refines this by showing how pH modulates the negative effect of cell density. Thus, although the 2FI model has predictive limitations, it successfully translates the biochemical principle of nutrient‐mediated repression into an intuitive process map, identifying the narrow cultivation window where B6 production becomes feasible.

#### 3.2.7 Pelletization

**FIGURE 8:**
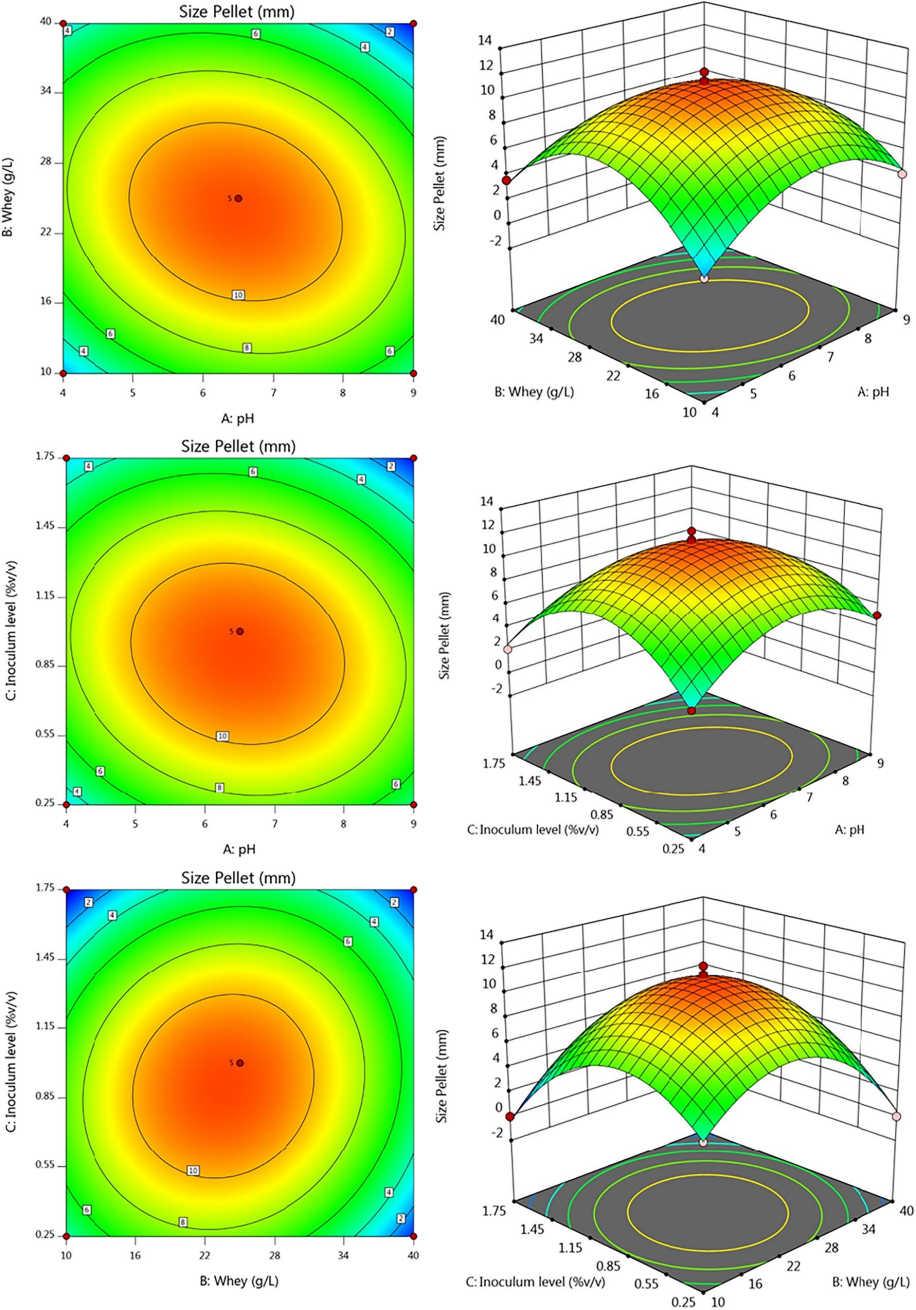
Response surface analysis illustrating the interactive effects of process variables on size pellet (mm) by *Neurospora intermedia* in whey medium at 30°C and 150 rpm.

The quadratic model for pellet size in *N*. *intermedia* demonstrated exceptional statistical robustness with a highly significant *F* value (98.78, *p* < 0.0001) and outstanding explanatory power, accounting for 99.22% of variance (*R* ^2^ = 0.9922) (Table S8), whereas a nonsignificant lack of fit (*p* = 0.5299) and close agreement between predicted *R* ^2^ (0.9425) and adjusted *R* ^2^ (0.9821) confirmed its reliability for both explanation and prediction. Factor analysis identified inoculum level (*C*) as the most influential linear factor (*p* = 0.0007, coefficient −1.275), followed by whey concentration (*B*) (*p* = 0.0072, coefficient −0.825), both exerting negative effects on pellet size, whereas pH (*A*) showed no significant linear influence. Remarkably, all two‐factor interactions (*AB*, *AC*, *BC*) and all quadratic terms (*A* ^2^, *B* ^2^, *C* ^2^) were highly significant (*p* < 0.05), with the quadratic terms exhibiting the most profound effects (*p* < 0.0001) through large negative coefficients (−3.6525, −5.2025, and −5.0525, respectively). The complete Equation (7) defines a strongly concave response surface, mathematically predicting that pellet size is maximized at specific intermediate values of all three factors near the design center and decreases symmetrically as factors deviate from these optima, providing a powerful quantitative tool for precise morphological control in this system (Figure 8):

(7) $$\begin{array}{ccc} & & \mathrm{Size}\mathrm{pellet}\;=11.28\;-\;0.2A\;-\;0.825B\;-\;1.275C\;-\;1.425AB\;-\\ & & \;1.025AC+1.025BC\;-\;3.6525A^{2}\;-\;5.2025B^{2}\;-\;5.0525C^{2} \end{array}$$

**FIGURE 9:**
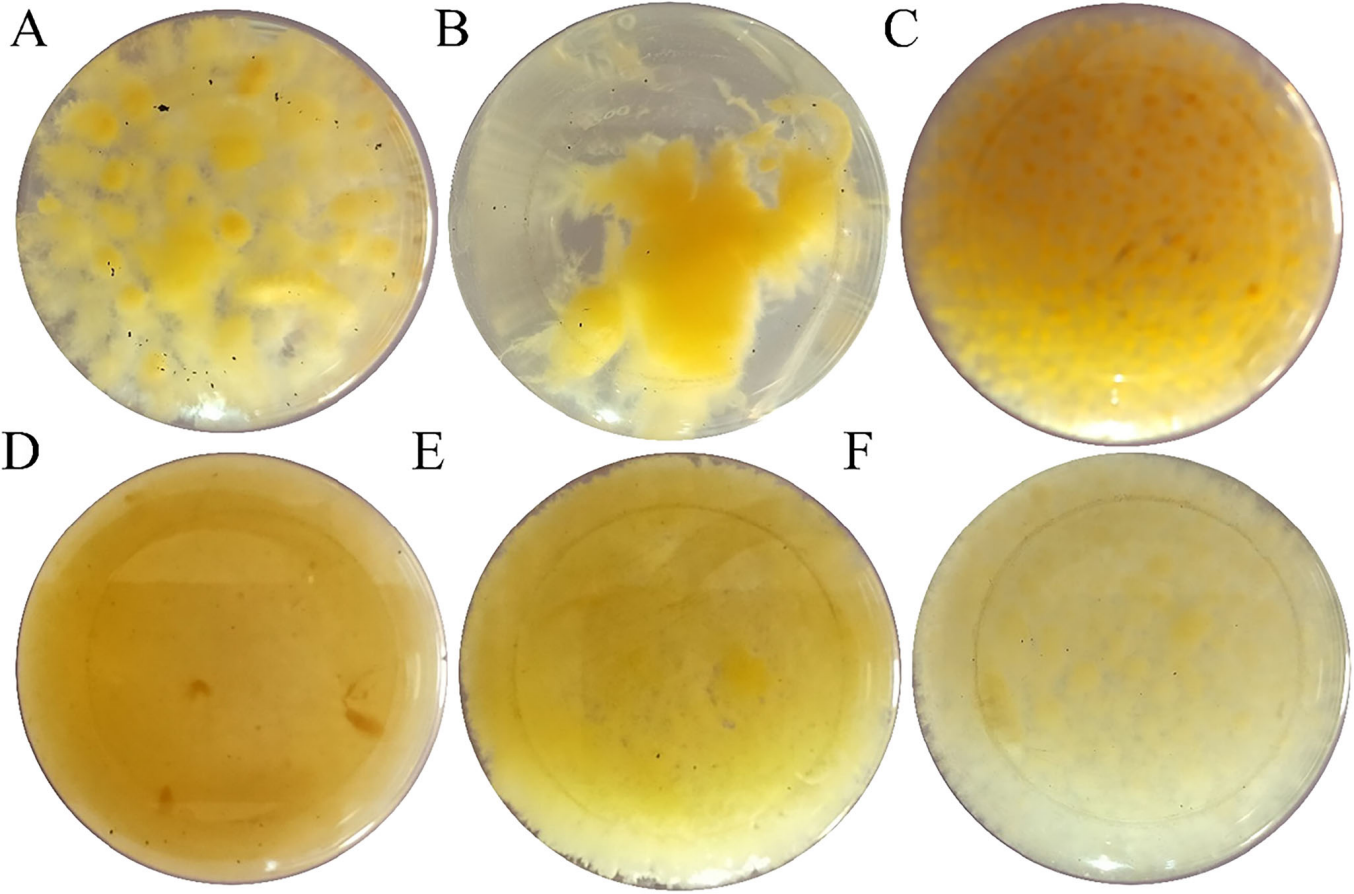
Different types of pellets *Neurospora intermedia*: (A) and (F) medium pellet (3–5 mm), (B) large pellet (10–12 mm), (C) small pellet (2–3 mm), (D) cohesive coil (0 mm), and (E) disjoint coil (0 mm).

The morphology of *N*. *intermedia* pellets is governed by a nonlinear response surface, where maximal pellet size occurs under balanced, substrate‐sufficient growth that favors radial hyphal extension. In contrast, minimized pellet size results from physiological shifts induced by strategic stresses like carbon starvation or pH deviations, which limit coordinated growth. These metabolic effects are significantly modulated by population density, as high inoculum levels amplify stress, potentially forcing a transition to dispersed filamentous growth. The resulting morphological spectrum, as illustrated in Figure 9, ranges from well‐defined spherical pellets of varying sizes (2–12 mm) to non‐pelleted coils. Notably, the smallest pellets (∼2 mm), while linked to reduced biomass yield, enhanced the production of carotenoids and B vitamins. This suggests that a compact morphology improves nutrient and oxygen accessibility per unit biomass, favoring secondary metabolite biosynthesis. Therefore, an inverse relationship exists between pellet size and metabolite production, highlighting morphology as a key process lever. Ultimately, product specificity in fungal fermentations is optimized by manipulating the interplay between nutrient availability, environmental stress, and quorum‐dependent physiological responses. Pellet formation was monitored because it critically affects downstream processing efficiency, as compact pellets of 2–5 mm diameter enable rapid gravity settling or simple sieving for biomass separation, reducing centrifugation costs by an estimated 40%–60% compared to filamentous dispersed growth (García‐Reyes et al. 2017).

**FIGURE 10:**
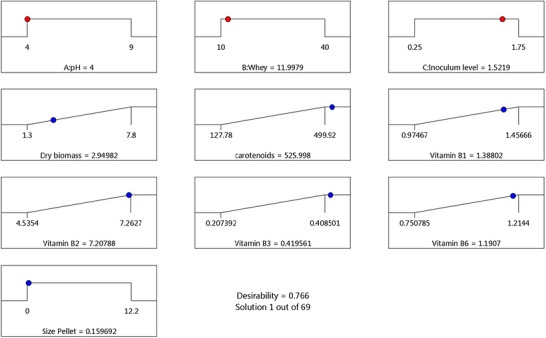
Desirability graph for the production of biomass, carotenoids, and vitamins B1, B2, B3, and B6 by *Neurospora intermedia*.

The desirability ramp graph indicates that *N*. *intermedia* can produce biomass, carotenoids, vitamins B1, B2, B3, and B6 up to 2.95 gDM/L, 525.99 µg/g, 1.39 mg/g, 7.21 mg/g, 0.42 mg/g, and 1.19 mg/g, respectively, under optimal conditions. These include a pH of 4, a whey concentration of 11.99 g/L, and an inoculum level of 1.53%, with 5 days of incubation at 30°C (Figure 10).

Downstream processing strategies for edible filamentous fungi include cell disruption, food‐grade solvent extraction, aqueous two‐phase systems, and integrated biorefining to recover vitamins and carotenoids. However, the whole fungal biomass itself is a high‐quality food and feed ingredient, providing naturally bioavailable compounds without requiring purification. When consumed intact (e.g., as mycoprotein or in fermented products), the native matrix enhances stability and intestinal delivery, eliminating costly processing and waste. Therefore, direct biomass use is recommended for most applications, with extraction reserved only for purified nutraceuticals.

The advantage of co‐production lies in operational flexibility, enabling three distinct RSM‐derived modes based on market demands. Mode 1 (mycoprotein‐maximizing: 40 g/L whey, pH 4) produces 7.8 g/L biomass with moderate nutraceuticals, optimal when bulk protein exceeds $3/kg. Mode 2 (nutraceutical‐maximizing: 10 g/L whey, pH 4) yields 1.7 g/L biomass with high‐value compounds (500 µg/g carotenoids, 7.2 mg/g B2), optimal when extracts exceed $100/kg. Mode 3 (morphology‐controlled: 25 g/L whey, pH 4, 0.25% inoculum) produces 2 mm pellets for low‐cost separation.

### 3.3 Reduction Metals, HPLC Sugar, and COD in Whey by Cultivation of Fungus

Fungal cultivation reduced the COD of the whey by 25.82%, from 3974 to 2948 mg/L, demonstrating effective biodegradation of its organic load. ICP‐OES analysis revealed substantial biosorption of metal ions. The process reduced concentrations of Ca, Mg, Fe, and K from initial values of 387.5, 83.1, 12.0, and 870 to 90, 25, 2.3, and 326 mg/L, respectively. This corresponds to high removal efficiencies of 76.8%, 69.9%, 80.8%, and 62.5% for Ca, Mg, Fe, and K. Given a dry biomass yield of 3.86 g/L, the specific metal uptake capacities were calculated as 77.1, 15.1, 2.51, and 140.9 mg/g of biomass for Ca, Mg, Fe, and K. This extensive uptake is consistent with the physiological demand for these essential elements: Mg^2^^+^ and K^+^ as enzyme cofactors and electrolytic balancers, and Ca^2^^+^ and Mg^2^^+^ as structural and signaling components. This assimilation reduced residual metal concentrations to just 19%–37% of their initial levels. This integrated process demonstrates a compelling dual benefit: the valorization of whey into fungal biomass coupled with efficient bioremediation and nutrient recovery. It represents a practical step toward a circular bioeconomy by closing nutrient loops and reducing the environmental impact of dairy wastewater. Kaya et al. (2025) reported that *N*. *intermedia* cultivated on whey bioaccumulated the following elements, including 5.24 g/kg Ca, 14.92 g/kg K, 6.55 g/kg Na, 964.14 mg/kg Mg, 228.93 mg/kg Fe, 71.32 mg/kg Cr, 40.57 mg/kg Zn, 11.91 mg/kg Cu, and 10.21 mg/kg Mn.

In the culture medium containing 25 g/L whey powder, *N*. *intermedia* was cultivated, and the lactose concentration decreased from 11.37 to 8.75 g/L, indicating that 23.04% of the initial lactose was consumed. The glucose concentration after cultivation reached 0.14 g/L, which was likely produced as a result of lactose hydrolysis during fungal growth. Similarly, Mahboubi et al. (2017b) indicated that *N*. *intermedia* were able to consume 29% of the lactose from cheese whey using *N*. *intermedia*.

### 3.4 Elemental Mass Balance and CHNS Transfer Efficiency From Cheese Whey to *Neurospora intermedia* Mycoprotein

CHNS analysis revealed a 2.24‐fold nitrogen enrichment from cheese whey (2.69% N) to *N*. *intermedia* biomass (6.04% *N*), corresponding to 37.8% crude protein (N × 6.25). The atomic C/N ratio was 7.84, which falls within the optimal range for actively growing filamentous fungi (5–15), confirming harvest during logarithmic phase. This protein content is comparable to *A*. *oryzae* grown on palm oil effluents (39.6%) and within the typical mycoprotein range (30%–50%). Our biomass yield of 4.5 g/L (at pH 4, 25 g/L whey) exceeded the 3.63 g/L reported by Kaya et al. (2024).

Whey contained only 0.16% sulfur, resulting in low fungal biomass sulfur (0.38% S) and a high atomic N/S ratio of 36.2. This ratio exceeds the high‐quality protein range (15–30), indicating severe deficiency in methionine and cysteine. The fungus cannot biosynthesize sulfur amino acids beyond the available sulfur pool, making this limitation substrate‐driven.

The atomic H/C ratio (1.85) and estimated C/O ratio (≈1.15) provide structural information about the biomass composition. The H/C ratio is notably higher than values reported for lignin‐rich biomass (H/C ≈ 1.0–1.2) and closely matches values for carbohydrate‐rich materials (glucose H/C = 2.0) and lipids (H/C ≈ 1.8–2.0). This indicates that *N*. *intermedia* biomass contains substantial amounts of storage carbohydrates (glycogen, trehalose) and cell wall polysaccharides (chitin, β‐glucans) in addition to protein. The C/O ratio of 1.15 is also consistent with polysaccharide‐rich biomass, as observed in other filamentous fungi (Table 4).

**TABLE 4:** Elemental composition (CHNS) of whey powder and fungal biomass cultivated on whey.

Sample	C (%)	H (%)	N (%)	S (%)	C/N ratio (atomic)	Protein (%) (N × 6.25)
Whey powder	36.76 ± 0.11	5.91 ± 0.04	2.69 ± 0.04	0.16 ± 0.02	15.9	16.81
Fungal biomass	40.45 ± 0.13	6.25 ± 0.06	6.04 ± 0.02	0.38 ± 0.01	7.8	37.75

*Note*: Values are presented as mean ± standard deviation (%). C/N atomic ratio was calculated as (C/12)/(N/14). Crude protein content was estimated using the conversion factor of 6.25.

## 4 Conclusion

Although preliminary OFAT optimization identified a single compromised condition (25 g/L whey, pH 7) for moderate outputs, subsequent RSM revealed this to be suboptimal, as OFAT failed to account for critical factor interactions. The RSM model instead delineated three distinct superior optima: maximum biomass yield (7.8 g/L) under high‐substrate, acidic conditions (40 g/L, pH 4); maximum intracellular carotenoids and B vitamins under low‐substrate, acidic conditions (10 g/L, pH 4); and conditions to minimize fungal pellet size to 2 mm (25 g/L, pH 4, 0.25% inoculum) for morphological control. This direct contrast between the first two optima highlights a critical substrate‐driven trade‐off between biomass accumulation and secondary metabolite synthesis. Collectively, the results demonstrate the fundamental importance of interacting factors (pH × whey × inoculum), undetectable by OFAT, in governing this bioprocess, with RSM providing the necessary framework for targeted optimization of biomass, metabolite‐rich biomass, or controlled morphology within a whey‐based biorefinery. The RSM models were developed within specific experimental boundaries (pH 4–9, whey 10–40 g/L, inoculum 0.25%–1.75%), and predictions outside these ranges remain untested. The OFAT screening cannot detect interaction effects. Future studies should expand the design space, employ more sensitive analytical techniques such as LC–MS/MS, and validate conditions in alternative cultivation modes.

## Author Contributions

**Farzaneh Dianatdar**: writing – review and editing, writing – original draft, methodology, investigation, formal analysis, data curation, conceptualization. **Zahra Etemadifar**: writing – review and editing, supervision, project administration, methodology, funding acquisition, conceptualization. **Mohammad J. Taherzadeh**: writing – review and editing, supervision, resources, methodology, conceptualization.

## Funding

We express our sincere gratitude to the University of Isfahan for providing financial support for this research endeavor. Additionally, this work is grounded in research funded by the Iran National Science Foundation (INSF) under project number 4027892.

## Conflicts of Interest

The authors declare no conflicts of interest.

## Supporting information




**Supplementary Materials**: jfds71294‐sup‐0001‐SuppMat.docx
