# Supplementary material for: Co‐Production of Mycoprotein, Carotenoids, and B Vitamins From Cheese Whey by Neurospora intermedia
Source: J Food Sci. 2026 Jul 23;91(7):e71294. doi: 10.1111/1750-3841.71294 (PMC13392596; doi:10.1111/1750-3841.71294)
Supplement: Supplementary file 1 — Supplementary Materials: jfds71294‐sup‐0001‐SuppMat.docx [file JFDS-91-0-s001.docx]

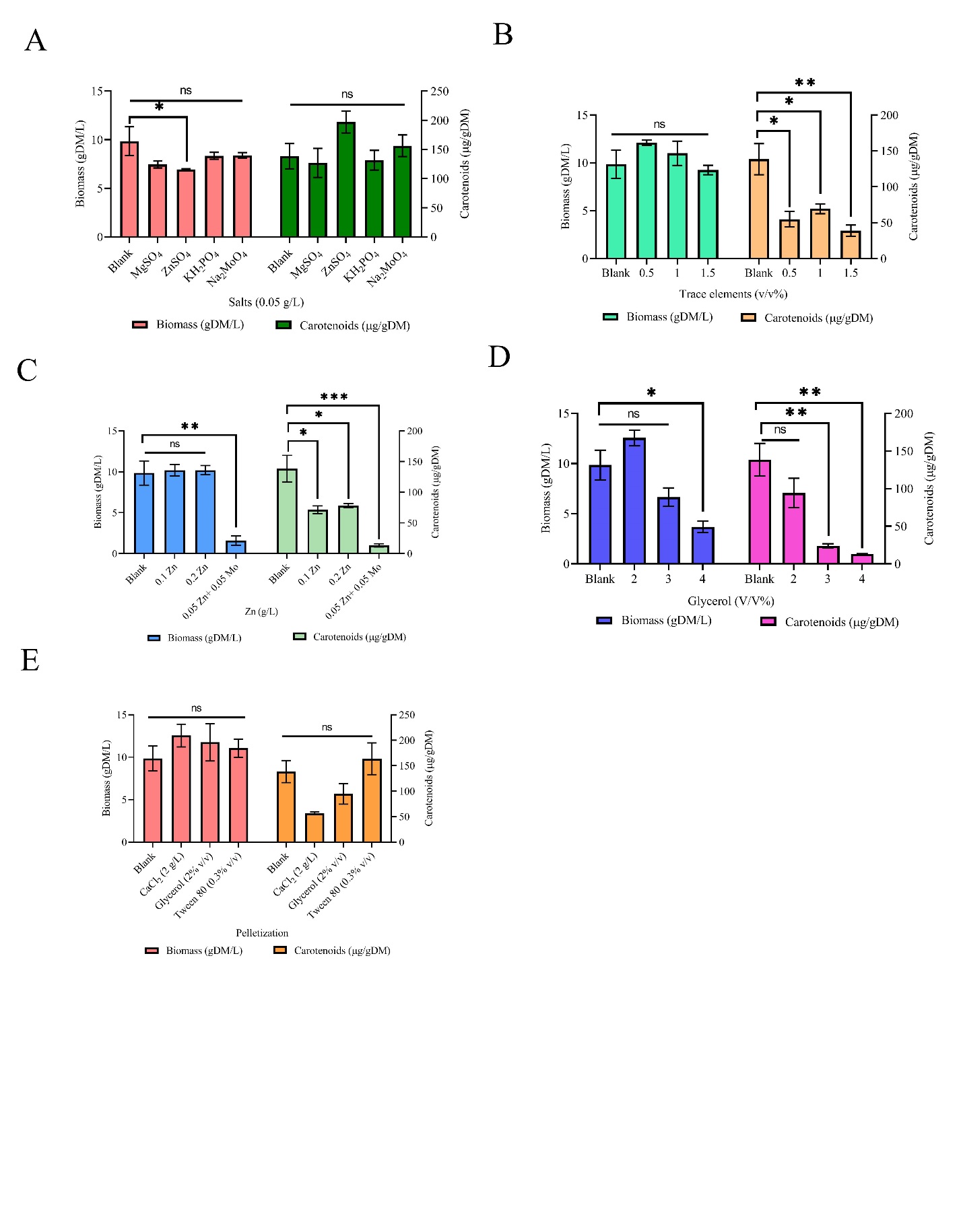


Fig. S 1. Effect of salts, trace elements, and pellet-inducing agents on biomass and carotenoid production by N. intermedia.


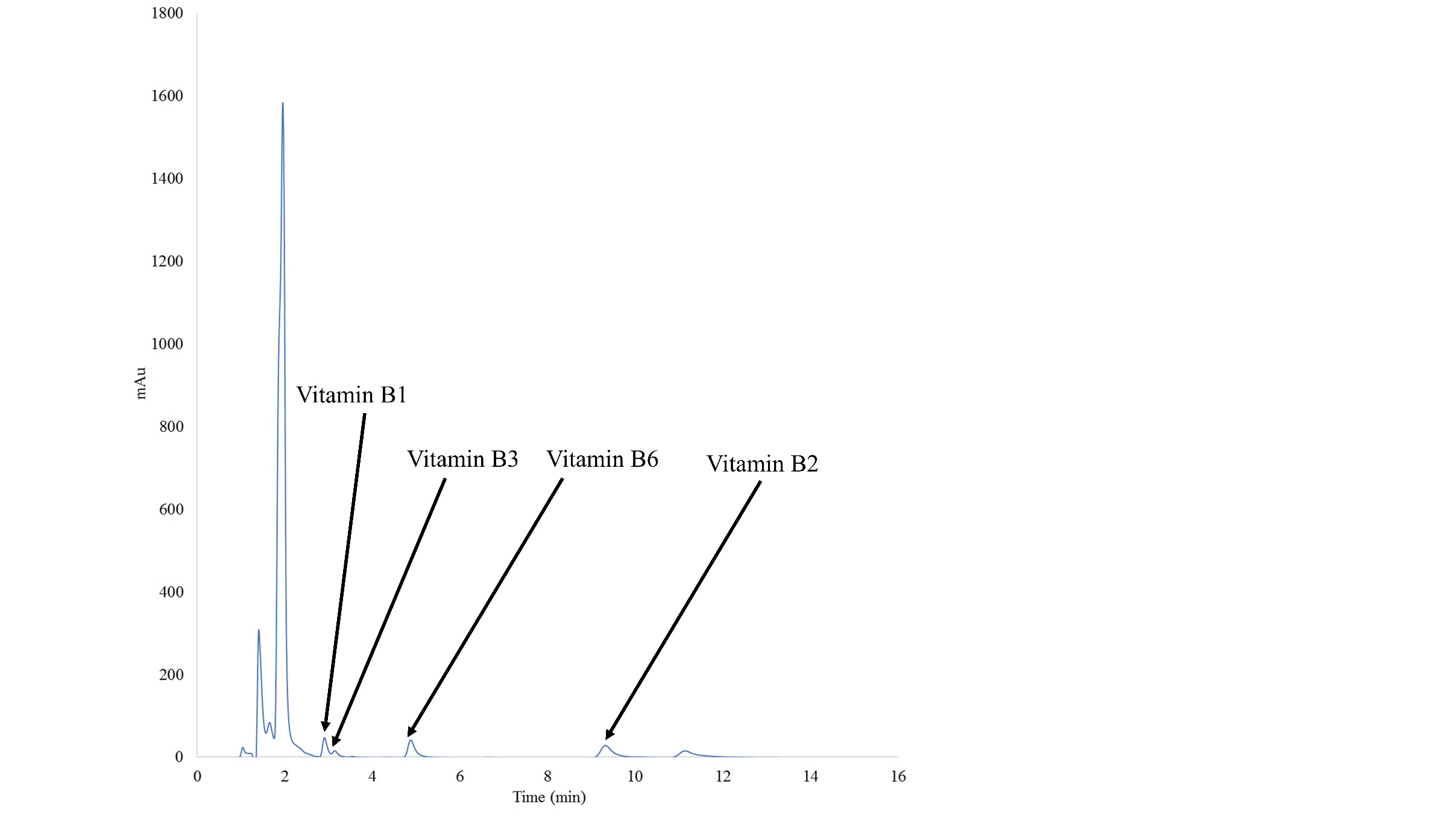


Fig. S 2. The HPLC analysis of vitamins B1, B2, B3, and B6 produced by N. intermedia was conducted in whey medium (1% v/v inoculum, 25 g/L whey, pH 7, 30°C, 150 rpm, 5 days).


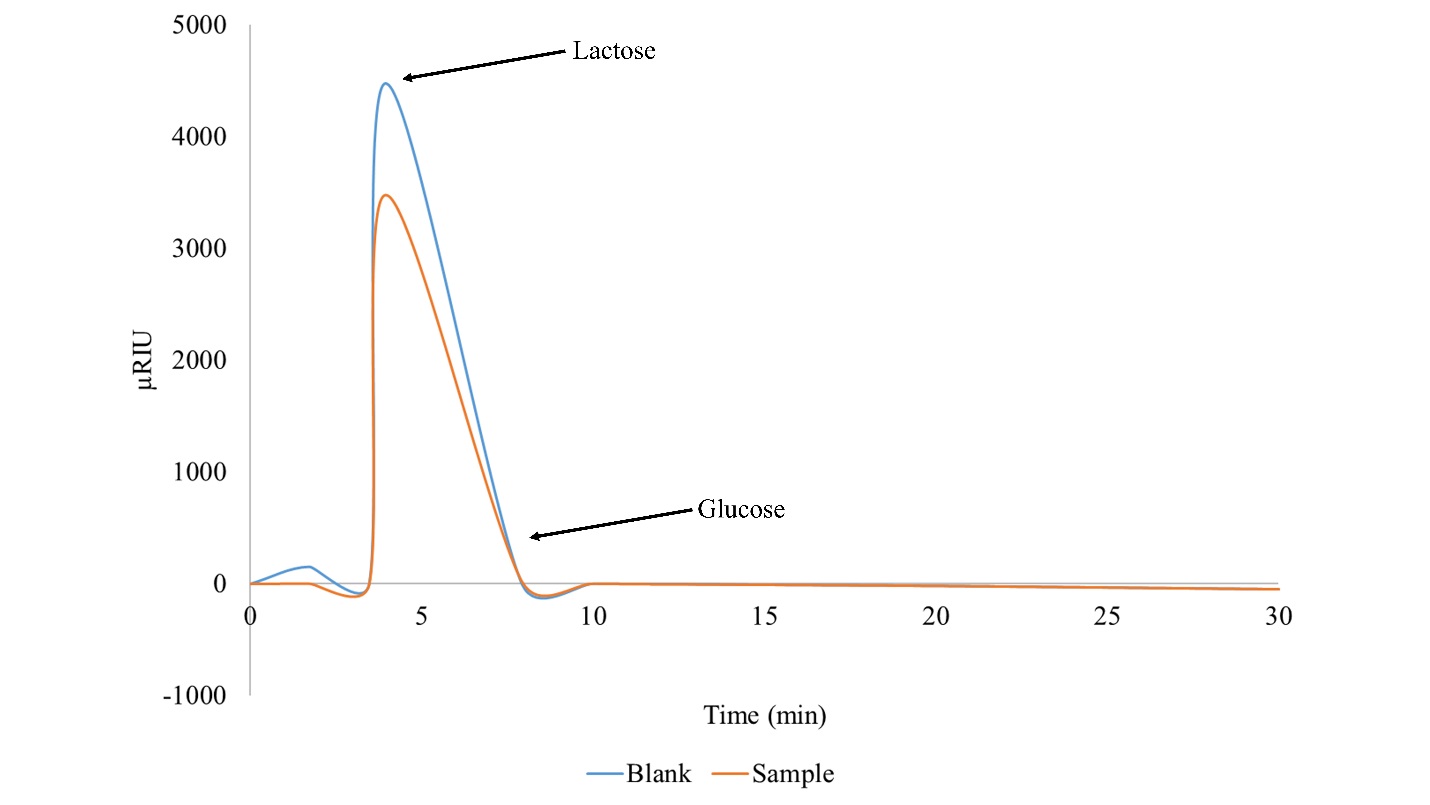


Fig. S 3. HPLC analysis lactose and glucose concentrations during cultivation of Neurospora intermedia in cheese whey medium (25 g/L initial whey powder).

Table S 1. Impact of salts, trace elements, and pellet-inducing agents on B‑vitamin production by N. intermedia on the yields of vitamins B1, B2, B3, and B6.

| Factors | | Vitamin B1 ± S.D (P-Value) | Vitamin B2 ± S.D (P-Value) | Vitamin B3 ± S.D (P-Value) | Vitamin B6 ± S.D (P-Value) |
| --- | --- | --- | --- | --- | --- |
| Metal | Blank^a^ | 1.66 ± 0.17 | 9.49 ± 0.3 | 0.26 ± 0.01 | 1.75 ± 0.04 |
|  | MgSO_4_ | 2.07 ± 0.22^N^ | 11.04 ± 0.33^N^ | 0.30 ± 0.01^N^ | 2.2 ± 0.33^N^ |
|  | ZnSO_4_ | 2 ± 0.07^N^ | 10.18 ± 0.1^N^ | 0.27 ± 0.01^N^ | 2.18 ± 0.04^N^ |
|  | KH_2_PO_4_ | 1.65 ± 0.28^N^ | 10.16 ± 0.07^N^ | 0.27 ± 0^N^ | 1.67 ± 0.32^N^ |
|  | Na_2_MoO_4_ | 1.48 ± 0.08^N^ | 9.95 ± 1.31^N^ | 0.26 ± 0.05^N^ | 1.41 ± 0.1^N^ |
| Zn+Mo | Blank^a^ | 1.66 ± 0.17 | 9.49 ± 0.3 | 0.26 ± 0.01 | 1.75 ± 0.04 |
|  | 0.1 Zn | 1.46 ± 0.18^N^ | 8.71 ± 1.04^N^ | 0.22 ± 0.01^N^ | 1.45 ± 0.02^N^ |
|  | 0.2 Zn | 1.19 ± 0.1^S^ | 9.61 ± 0.4^N^ | 0.24 ± 0.05^N^ | 1.37 ± 0.22^N^ |
|  | 0.05 Zn+0.05 Mo | 1.29 ± 0.04^N^ | 9.94 ± 0.51^N^ | 0.23 ± 0.01^N^ | 1.33 ± 0.13^N^ |
| Trace elements (v/v%) | Blank^a^ | 1.66 ± 0.17 | 9.49 ± 0.3 | 0.26 ± 0.01 | 1.75 ± 0.04 |
|  | 0.5 | 1.38 ± 0.05^N^ | 11.14 ± 0.04^N^ | 0.27 ± 0^N^ | 1.5 ± 0.04^S^ |
|  | 1 | 1.32 ± 0.03^N^ | 11.44 ± 0.38^N^ | 0.26 ± 0.04^N^ | 1.29 ± 0.02^S^ |
|  | 1.5 | 1.27 ± 0.15^S^ | 11.68 ± 0.5 ^N^ | 0.25 ± 0.01^N^ | 1.14 ± 0.1^S^ |
| Gly (v/v%) | Blank^a^ | 1.66 ± 0.17 | 9.49 ± 0.3 | 0.26 ± 0.01 | 1.75 ± 0.04 |
|  | 2 | 1.35 ± 0.01^N^ | 10.83 ± 1.01^N^ | 0.28 ± 0.03^N^ | 1.21 ± 0.08^S^ |
|  | 3 | 1.37 ± 0.13^N^ | 10.9 ± 0.31 ^N^ | 0.29 ± 0.01^N^ | 1.42 ± 0.11^S^ |
|  | 4 | 1.27 ± 0.1^N^ | 9.98 ± 0.91 ^N^ | 0.27 ± 0.01^N^ | 1.2 ± 0.04^S^ |

^a: whey 70 (g/L), pH 7, and 0.5 (g/L) KNO3^

^S: significant^

^N: non-significant^

Table S 2. ANOVA for the dried biomass (gDM/L) production by N. intermedia^a^.

| Source | Sum of Squares | df | Mean Square | F-value | p-value |  |
| --- | --- | --- | --- | --- | --- | --- |
| Model | 47.53 | 3 | 15.84 | 39.92 | < 0.0001 | significant |
| A-pH | 6.12 | 1 | 6.12 | 15.43 | 0.0017 |  |
| B-Whey | 41.41 | 1 | 41.41 | 104.31 | < 0.0001 |  |
| C-Inoculum level | 0.0050 | 1 | 0.0050 | 0.0126 | 0.9124 |  |
| Residual | 5.16 | 13 | 0.3969 |  |  |  |
| Lack of Fit | 4.71 | 9 | 0.5231 | 4.63 | 0.0771 | not significant |
| Pure Error | 0.4520 | 4 | 0.1130 |  |  |  |
| Cor Total | 52.70 | 16 |  |  |  |  |

^a: C.V. % = 15.64; R2 = 0.9021; Adjusted R2 = 0.8795; Predicted R2 = 0.8040; Adequate precision = 20.6143. Non-significant at p-value > 0.05, Significant at p-value < 0.05.^

Table S 3. ANOVA for the carotenoids (µg/g) production by N. intermedia^a^.

| Source | Sum of Squares | df | Mean Square | F-value | p-value |  |
| --- | --- | --- | --- | --- | --- | --- |
| Model | 45232.41 | 9 | 5025.82 | 34.51 | < 0.0001 | significant |
| A-pH | 16572.93 | 1 | 16572.93 | 113.81 | < 0.0001 |  |
| B-Whey | 11393.40 | 1 | 11393.40 | 78.24 | < 0.0001 |  |
| C-Inoculum level | 4114.79 | 1 | 4114.79 | 28.26 | 0.0011 |  |
| AB | 1868.61 | 1 | 1868.61 | 12.83 | 0.0089 |  |
| AC | 2090.09 | 1 | 2090.09 | 14.35 | 0.0068 |  |
| BC | 338.61 | 1 | 338.61 | 2.33 | 0.1711 |  |
| A² | 7839.13 | 1 | 7839.13 | 53.83 | 0.0002 |  |
| B² | 1119.86 | 1 | 1119.86 | 7.69 | 0.0276 |  |
| C² | 108.64 | 1 | 108.64 | 0.7461 | 0.4163 |  |
| Residual | 1019.32 | 7 | 145.62 |  |  |  |
| Lack of Fit | 866.05 | 3 | 288.68 | 7.53 | 0.0402 | significant |
| Pure Error | 153.26 | 4 | 38.32 |  |  |  |
| Cor Total | 46251.73 | 16 |  |  |  |  |

^a: C.V. % = 8.20; R2 = 0.9780; Adjusted R2 = 0.9496; Predicted R2 = 0.6952; Adequate precision = 18.8836. Significant at p-value < 0.05. Non-significant at p-value > 0.05.^

^Note: Insignificant terms (p > 0.05) were retained in the model to preserve hierarchical structure and prevent biased coefficient estimates. Backward elimination (p < 0.10 criterion) was tested and yielded nearly identical predictions (R² change <0.01), confirming that retention of insignificant terms does not compromise model utility.^

Table S 4. ANOVA for the B1 vitamin (mg/g) production by N. intermedia^a^.

| Source | Sum of Squares | df | Mean Square | F-value | p-value |  |
| --- | --- | --- | --- | --- | --- | --- |
| Model | 125.32 | 9 | 13.92 | 105.56 | < 0.0001 | significant |
| A-pH | 0.0009 | 1 | 0.0009 | 0.0066 | 0.9375 |  |
| B-Whey | 111.41 | 1 | 111.41 | 844.58 | < 0.0001 |  |
| C-Inoculum level | 0.0061 | 1 | 0.0061 | 0.0463 | 0.8357 |  |
| AB | 0.4929 | 1 | 0.4929 | 3.74 | 0.0945 |  |
| AC | 0.9056 | 1 | 0.9056 | 6.87 | 0.0344 |  |
| BC | 0.1821 | 1 | 0.1821 | 1.38 | 0.2784 |  |
| A² | 0.0459 | 1 | 0.0459 | 0.3483 | 0.5736 |  |
| B² | 1.15 | 1 | 1.15 | 8.75 | 0.0212 |  |
| C² | 10.56 | 1 | 10.56 | 80.09 | < 0.0001 |  |
| Residual | 0.9233 | 7 | 0.1319 |  |  |  |
| Lack of Fit | 0.5539 | 3 | 0.1846 | 2.00 | 0.2565 | not significant |
| Pure Error | 0.3694 | 4 | 0.0924 |  |  |  |
| Cor Total | 126.24 | 16 |  |  |  |  |

^a: C.V. % = 1.46; R2 = 0.9927; Adjusted R2 = 0.9833; Predicted R2 = 0.9252; Adequate precision = 34.1928. Significant at p-value < 0.05. Non-significant at p-value > 0.05.^

^Note: Insignificant terms (p > 0.05) were retained in the model to preserve hierarchical structure and prevent biased coefficient estimates. Backward elimination (p < 0.10 criterion) was tested and yielded nearly identical predictions (R² change <0.01), confirming that retention of insignificant terms does not compromise model utility.^

Table S 5. ANOVA for the B2 vitamin (mg/g) production by N. intermedia^a^.

| Source | Sum of Squares | df | Mean Square | F-value | p-value |  |
| --- | --- | --- | --- | --- | --- | --- |
| Model | 5410.74 | 9 | 601.19 | 53.83 | < 0.0001 | significant |
| A-pH | 17.69 | 1 | 17.69 | 1.58 | 0.2486 |  |
| B-Whey | 4588.73 | 1 | 4588.73 | 410.85 | < 0.0001 |  |
| C-Inoculum level | 0.1732 | 1 | 0.1732 | 0.0155 | 0.9044 |  |
| AB | 0.8617 | 1 | 0.8617 | 0.0772 | 0.7892 |  |
| AC | 30.33 | 1 | 30.33 | 2.72 | 0.1434 |  |
| BC | 0.4880 | 1 | 0.4880 | 0.0437 | 0.8404 |  |
| A² | 384.16 | 1 | 384.16 | 34.40 | 0.0006 |  |
| B² | 11.40 | 1 | 11.40 | 1.02 | 0.3461 |  |
| C² | 319.41 | 1 | 319.41 | 28.60 | 0.0011 |  |
| Residual | 78.18 | 7 | 11.17 |  |  |  |
| Lack of Fit | 39.00 | 3 | 13.00 | 1.33 | 0.3829 | not significant |
| Pure Error | 39.18 | 4 | 9.80 |  |  |  |
| Cor Total | 5488.92 | 16 |  |  |  |  |

^a: C.V. % = 2.87; R2 = 0.9858; Adjusted R2 = 0.9674; Predicted R2 = 0.8752; Adequate precision = 19.8475. Significant at p-value < 0.05. Non-significant at p-value > 0.05.^

^Note: Insignificant terms (p > 0.05) were retained in the model to preserve hierarchical structure and prevent biased coefficient estimates. Backward elimination (p < 0.10 criterion) was tested and yielded nearly identical predictions (R² change <0.01), confirming that retention of insignificant terms does not compromise model utility.^

Table S 6. ANOVA for the B3 vitamin (mg/g) production by N. intermedia^a^.

| Source | Sum of Squares | df | Mean Square | F-value | p-value |  |
| --- | --- | --- | --- | --- | --- | --- |
| Model | 7.20 | 9 | 0.7999 | 22.30 | 0.0002 | significant |
| A-pH | 0.3312 | 1 | 0.3312 | 9.23 | 0.0189 |  |
| B-Whey | 4.84 | 1 | 4.84 | 135.00 | < 0.0001 |  |
| C-Inoculum level | 0.0084 | 1 | 0.0084 | 0.2339 | 0.6434 |  |
| AB | 0.1846 | 1 | 0.1846 | 5.15 | 0.0576 |  |
| AC | 0.1146 | 1 | 0.1146 | 3.20 | 0.1170 |  |
| BC | 0.0004 | 1 | 0.0004 | 0.0121 | 0.9155 |  |
| A² | 1.18 | 1 | 1.18 | 32.88 | 0.0007 |  |
| B² | 0.1756 | 1 | 0.1756 | 4.90 | 0.0626 |  |
| C² | 0.2232 | 1 | 0.2232 | 6.22 | 0.0413 |  |
| Residual | 0.2511 | 7 | 0.0359 |  |  |  |
| Lack of Fit | 0.2162 | 3 | 0.0721 | 8.25 | 0.0345 | significant |
| Pure Error | 0.0349 | 4 | 0.0087 |  |  |  |
| Cor Total | 7.45 | 16 |  |  |  |  |

^a: C.V. % = 6.28; R2 = 0.9663; Adjusted R2 = 0.9230; Predicted R2 = 0.5285; Adequate precision = 13.6698. Significant at p-value < 0.05. Non-significant at p-value > 0.05.^

^Note: Insignificant terms (p > 0.05) were retained in the model to preserve hierarchical structure and prevent biased coefficient estimates. Backward elimination (p < 0.10 criterion) was tested and yielded nearly identical predictions (R² change <0.01), confirming that retention of insignificant terms does not compromise model utility.^

Table S 7. ANOVA for the B6 vitamin (mg/g) production by N. intermedia^a^.

| Source | Sum of Squares | df | Mean Square | F-value | p-value |  |
| --- | --- | --- | --- | --- | --- | --- |
| Model | 86.92 | 6 | 14.49 | 6.96 | 0.0040 | significant |
| A-pH | 0.0202 | 1 | 0.0202 | 0.0097 | 0.9235 |  |
| B-Whey | 21.33 | 1 | 21.33 | 10.25 | 0.0095 |  |
| C-Inoculum level | 39.48 | 1 | 39.48 | 18.97 | 0.0014 |  |
| AB | 7.48 | 1 | 7.48 | 3.60 | 0.0871 |  |
| AC | 3.10 | 1 | 3.10 | 1.49 | 0.2501 |  |
| BC | 15.50 | 1 | 15.50 | 7.45 | 0.0212 |  |
| Residual | 20.81 | 10 | 2.08 |  |  |  |
| Lack of Fit | 14.45 | 6 | 2.41 | 1.52 | 0.3577 | not significant |
| Pure Error | 6.35 | 4 | 1.59 |  |  |  |
| Cor Total | 107.72 | 16 |  |  |  |  |

^a: C.V. % = 6.90; R2 = 0.8068; Adjusted R2 = 0.6910; Predicted R2 = 0.3218; Adequate precision = 9.5878. Significant at p-value < 0.05. Non-significant at p-value > 0.05.^

^Note: Insignificant terms (p > 0.05) were retained in the model to preserve hierarchical structure and prevent biased coefficient estimates. Backward elimination (p < 0.10 criterion) was tested and yielded nearly identical predictions (R² change <0.01), confirming that retention of insignificant terms does not compromise model utility.^

Table S 8. ANOVA for the size pellet (mm) production by N. intermedia^a^.

| Source | Sum of Squares | df | Mean Square | F-value | p-value |  |
| --- | --- | --- | --- | --- | --- | --- |
| Model | 344.55 | 9 | 38.28 | 98.78 | < 0.0001 | significant |
| A-pH | 0.3200 | 1 | 0.3200 | 0.8257 | 0.3937 |  |
| B-Whey | 5.44 | 1 | 5.44 | 14.05 | 0.0072 |  |
| C-Inoculum level | 13.00 | 1 | 13.00 | 33.56 | 0.0007 |  |
| AB | 8.12 | 1 | 8.12 | 20.96 | 0.0026 |  |
| AC | 4.20 | 1 | 4.20 | 10.84 | 0.0132 |  |
| BC | 4.20 | 1 | 4.20 | 10.84 | 0.0132 |  |
| A² | 56.17 | 1 | 56.17 | 144.93 | < 0.0001 |  |
| B² | 113.96 | 1 | 113.96 | 294.04 | < 0.0001 |  |
| C² | 107.49 | 1 | 107.49 | 277.33 | < 0.0001 |  |
| Residual | 2.71 | 7 | 0.3876 |  |  |  |
| Lack of Fit | 1.06 | 3 | 0.3550 | 0.8617 | 0.5299 | not significant |
| Pure Error | 1.65 | 4 | 0.4120 |  |  |  |
| Cor Total | 347.26 | 16 |  |  |  |  |

^a: C.V. % = 13.15; R2 = 0.9922; Adjusted R2 = 0.9821; Predicted R2 = 0.9425; Adequate precision = 24.5667. Significant at p-value < 0.05. Non-significant at p-value P > 0.05.^

^Note: Insignificant terms (p > 0.05) were retained in the model to preserve hierarchical structure and prevent biased coefficient estimates. Backward elimination (p < 0.10 criterion) was tested and yielded nearly identical predictions (R² change <0.01), confirming that retention of insignificant terms does not compromise model utility.^
